# Highly Selective Exogenous Neutrophils Effectively Inhibit Growth of Colon Cancer under the Guidance of Precision Navigation

**DOI:** 10.34133/research.0894

**Published:** 2025-09-16

**Authors:** Yunxi Yang, Chuyu Li, Yiming Shao, Xiao Wen, Cheng Lu, Xi Gao, Yiwen Mei, Bingwei Sun

**Affiliations:** ^1^ Research Center for Neutrophil Engineering Technology, The Affiliated Suzhou Hospital of Nanjing Medical University, Suzhou 215002, Jiangsu Province, China.; ^2^ State Key Laboratory of Reproductive Medicine, Center of Clinical Reproductive Medicine, The First Affiliated Hospital of Nanjing Medical University, Jiangsu Province Hospital, Nanjing 210029, China.

## Abstract

The diversity of neutrophils’ subtypes and functions in the tumor microenvironmentunderlies their contributions to both pro- and anti-tumorigenic activities. How to elucidate the antitumor mechanisms of neutrophil and effectively utilize it still needs to be studied. In this study, neutrophil stimulus reaction index (NSRI) was established for high selectivity of neutrophils. Sufficient and highly selective exogenous neutrophils were used to infuse into tumor-bearing mice. Navigation technique was used to make neutrophils rapidly infiltrate into the tumor tissue, which played a key role in inhibiting tumor growth. We found that tumor cells were efficiently killed in direct coculture with sufficient neutrophils by promoting a multimodal death primarily dominated by apoptosis. Interestingly, colon cancer organoid diameters were decreased markedly cocultured with neutrophils, and were more remarkable in the application of organoid microinjection of neutrophils. Consistent with the 3D-printed model, in vivo model indicated that neutrophils meaningfully inhibited the tumor growth by formation of neutrophil extracellular traps (NETs) with neutrophil elastase (NE) payload. This study not only unveils the crucial anticancer effects of neutrophils but also sets out an innovative strategy of aggressive dose exogenous neutrophils with precise navigation, which provided the basis for advancements in neutrophil-based immunotherapy for colon cancer treatment.

## Introduction

Increasing evidence underscores the significant involvement of neutrophils across various stages of tumorigenesis [1,2]. However, the dynamic role of neutrophils in tumorigenesis remains incompletely understood, owing to their phenotypic plasticity and multifaceted biological functions [3,4]. Prevailing research portrays neutrophils primarily as contributors to tumor progression, fostering tumor cell proliferation [5,6], angiogenesis [7–9], and metastasis [10–12] within the tumor microenvironment. Notably, the biological function of neutrophils is closely linked to their microenvironmental context [13–15], activation status [16,17], and disease dynamics [18,19].

Elevated levels of peripheral blood neutrophils accompany cancer progression [20], with neutrophilic leukocytosis often correlating with poor prognosis in certain cancers [21,22]. Recent investigations have focused on tumor-associated neutrophils (TANs), revealing their dualistic roles as both inhibitors and promoters of tumor growth. Neutrophil extracellular traps (NETs) generated by neutrophils facilitate premetastatic niche formation, epithelial–mesenchymal transition, and interaction with circulating tumor cells, thereby contributing to tumor dissemination and distant colonization [23–25]. Moreover, the tumor microenvironment activates neutrophils and promotes the formation of low-density neutrophils, exhibiting characteristics such as immaturity and immunosuppression, influencing tumor cell behavior, signaling, and immune escape mechanisms [26,27]. However, conflicting evidence also suggests neutrophils’ potential for direct antitumor activity, such as reactive oxygen species (ROS) and reactive nitrogen species (RNS) generation [28], antibody-dependent cellular cytotoxicity (ADCC), and crosstalk with other immune effectors to enhance antitumor immune responses [29]. Consequently, divergent perspectives persist regarding the precise role of neutrophils in the tumor microenvironment, warranting further investigation into their biological behavior during tumorigenesis [30,31].

To address this scientific gap, the present study leverages an established neutrophil research platform to elucidate the role of a sufficient number of fresh neutrophils with precise navigation during early colon cancer development. Neutrophil stimulus reaction index (NSRI) was preliminary established for high selection of neutrophils to ensure cells with efficient immune function were screened for studies. Specifically, through in vitro coculture experiments with colon cancer cells, colon cancer organoid neutrophil micro-injection, and 3-dimensional (3D)-printed tumor microenvironment models, we aim to delineate the conditions conducive to neutrophil-mediated tumor cell killing. Additionally, the morphology, function, and apoptotic characteristics of neutrophils and colon cancer cells under these conditions were investigated to reveal the specific mechanism by which neutrophils kill colon cancer cells. in vivo experiments entailed the establishment of colon cancer-bearing mice model, followed by the construction of neutrophil clearance and aggressive dose of fresh neutrophil allogeneic transfusion (NAT) mouse models. Furthermore, the specific status of colon cancer tumor growth was observed in 2 extreme states, i.e., neutrophil-deficient and neutrophil-enriched. The induction of sufficient neutrophil infiltration within the tumor microenvironment was achieved through interleukin-8 (IL-8) peritumoral injection combined with NAT, facilitating dynamic observations of tumorigenesis and intratumoral neutrophil dynamics in mice, and the immune role of neutrophils in the tumor microenvironment was explored, highlighting neutrophils with precise navigation as promising effectors for tumor immunotherapy.

## Results

### NETs, generated by neutrophils, adhering and affecting colon cancer cells

**Fig. 1.**
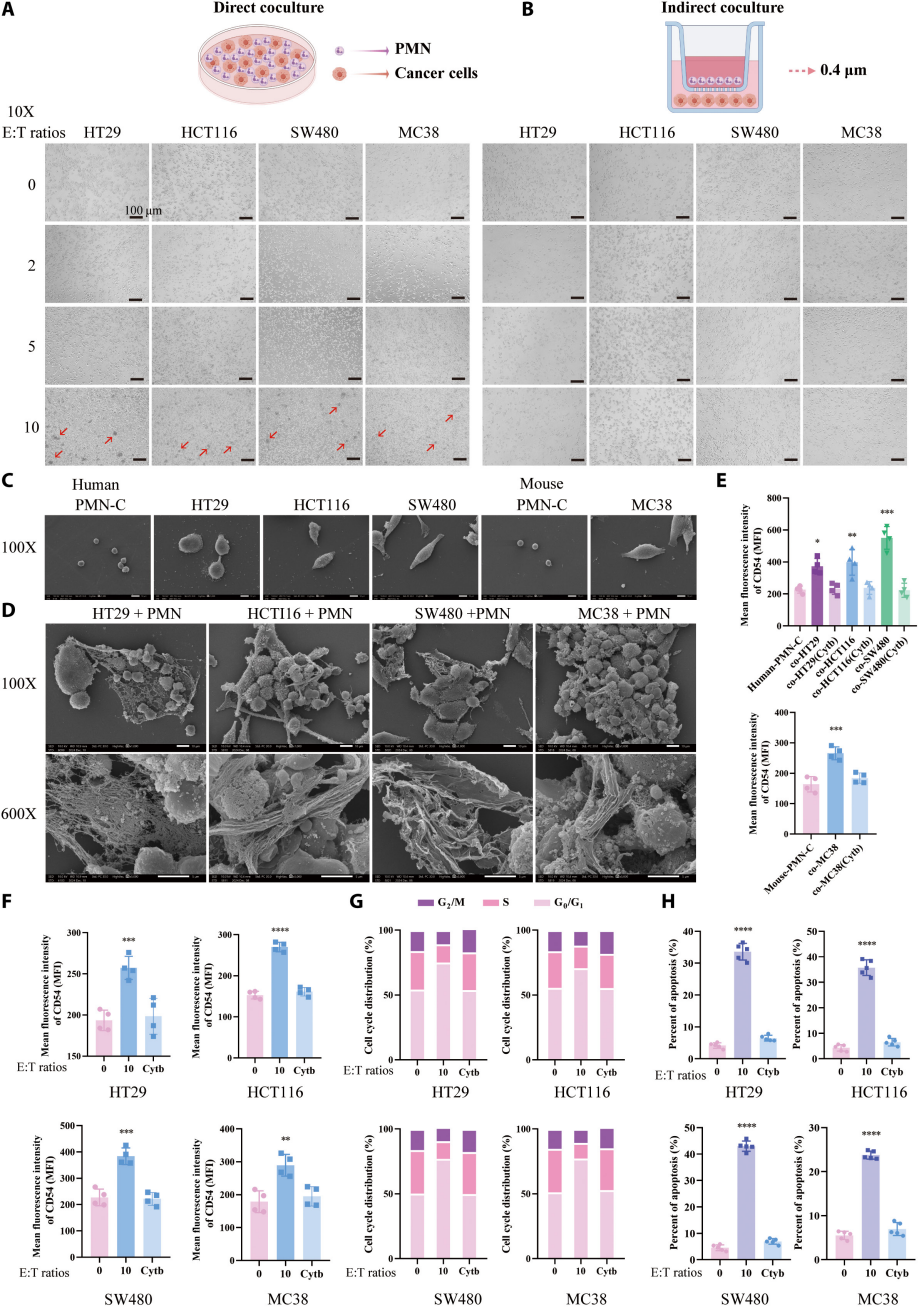
Generation of NETs and growth status of colon cancer cells after cocultured for 24 h. (A) Cell morphology of each group of neutrophils and colon cancer cells directly cocultured for 24 h. (B) Cell morphology of each group of neutrophils and colon cancer cells indirectly cocultured for 24 h. Scale bar, 100 μm. (C) SEM morphology of neutrophils and colon cancer cells under normal culture conditions. (D) SEM morphology of neutrophils and colon cancer cells after coculture. (E) Increased expression of CD54 in neutrophils after coculture. (F) Increased expression of CD54 in various colon cancer cells after coculture. (G) Cell cycle profiles of colon cancer cells after 24 h of coculture. (H) Apoptosis of colon cancer cells after 24 h of coculture. *P < 0.05, **P < 0.01, ***P < 0.001, ****P < 0.0001 compared to the control group.

Four colon cancer cells were chosen for this study—human cell lines (HT29, HCT116, and SW480) and murine cell lines (MC38). All the colon cancer cells grew well (Fig. S2). A significant decrease in colon cancer cell fusion was observed after 24 h of coculture with neutrophils, as seen using the OLYMPUS Cell Dynamic Assay Device (Movie S1). Thus, to explore the primary mode of tumor cell killing by neutrophils, both direct (Fig. 1A) and indirect cocultures (Fig. 1B) were conducted. The coculture experiments were divided into 3 ratios of neutrophils to colon cancer cells [effector cells:target cells (E:T)]: 2:1, 5:1, and 10:1. After 24 h of coculture, the direct coculture with 10 times the number of neutrophils exhibited significant cell cluster aggregation (Fig. 1A) and increased floating cells and the medium color turned yellow. However, the other groups did not exhibit significant changes in cell status. Therefore, these findings suggest that 2 conditions are required for neutrophil-mediated tumor cell killing: (a) direct contact and (b) a higher number of neutrophils. Subsequent direct coculture experiments were conducted with a 10-fold order of magnitude. Observation under scanning electron microscopy (SEM) of cells revealed that normally cultured neutrophils were spherical cells without extracellular fibers, and colon cancer cells exhibited an intact cytoskeleton and healthy growth (Fig. 1C). However, flattened neutrophils with numerous membrane protrusions and the formation of extracellular reticular fibers (NETs) were visible after coculture. The reticulated structure of the extruded NETs captured and encapsulated the colon cancer cells (Fig. 1D). Following coculture, the expression of intercellular adhesion molecule (ICAM-1, CD54) significantly increased in both neutrophils and various colon cancer cells (Fig. 1E and F and Fig. S3A), indicating stronger adhesion between neutrophils and colon cancer cells upon NET generation, creating a tightly interconnected system. Colon cancer cells, both human-derived (HT29, HCT116, and SW480 cells) and murine-derived (MC38), exhibited pronounced G_0_/G_1_ phase arrest after coculture (Fig. 1G and Fig. S3B), indicating that most tumor cells were in a cytostatic phase, unable to divide and proliferate. Prolonged G_0_/G_1_ phase arrest led to apoptosis. Further analysis confirmed a significant increase in apoptosis in all 4 types of colon cancer cells after 24 h of coculture with neutrophils (Fig. 1H and Fig. S3C). These phenomena were alleviated in the cytochalasin B (cytb) intervention group, in which neutrophils were pretreated with 100 nM cytb for 0.5 h for cytoskeleton stabling—intercellular adhesion was decreased, while apoptosis and cell cycle of tumor cells were not significantly affected, indicating the role mediated by NETs.

### Neutrophil induced GSDMD-mediated cell death in colon cancer cells

**Fig. 2.**
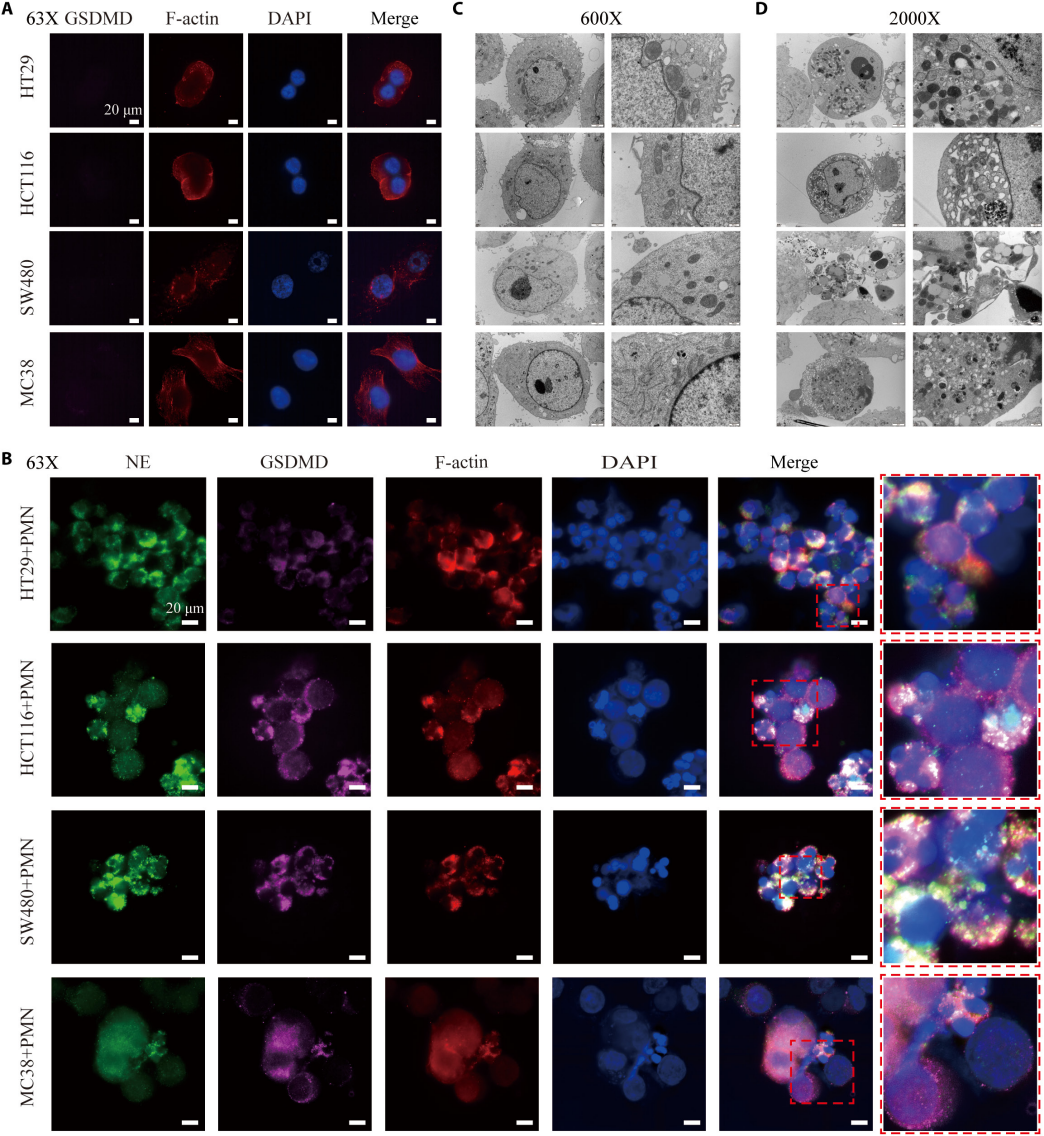
Increased expression of GSDMD-NT contributing to the death of colon cancer cells. (A) Expression of GSDMD-NT and F-actin in colon cancer cells under normal culture conditions. (B) Expression of GSDMD-NT, F-actin, and NET generation in coculture system. (C) Structural representation of colon cancer cells under the TEM. (D) Apoptotic manifestations of colon cancer cells after coculture. Scale bar, 20 μm.

The cleavage of gasdermin D (GSDMD) by intracellular NE generates the active N-terminal fragment (GSDMD-NT). This fragment then forms pores in the plasma and granular membranes of neutrophils, which is one of the main mechanism for NETs formation [32,33]. Structured illumination microscopy (SIM) imaging revealed low expression of GSDMD-NT in colon cancer cells (Fig. 2A). However, there was a significant increase in GSDMD-NT expression, accompanied by cytoskeletal disruption and nuclear volume reduction after coculture with neutrophils. GSDMD-NT forms pores on the plasma membrane of colon cancer cells, enabling NE (released with NETs) to enter the colon cancer cells and even penetrate into the nucleus, damaging and lysing the cells (Fig. 2B). Transmission electron microscopy (TEM) showed that, compared to normally cultured cells (Fig. 2C), the apoptotic manifestations of colon cancer cells emerged after coculture, such as volume reduction, cytoplasmic condensation, mitochondrial aggregation, chromatin agglutination, and even apoptotic bodies sprouted from the cell membranes (Fig. 2D). Additionally, pyroptosis and autophagy in various groups of colon cancer cells were observed (Fig. S4), indicating that the killing of colon cancer cells by neutrophils is a multimodal death primarily dominated by apoptosis.

### The successful construction and validation of colon cancer organoids

**Fig. 3.**
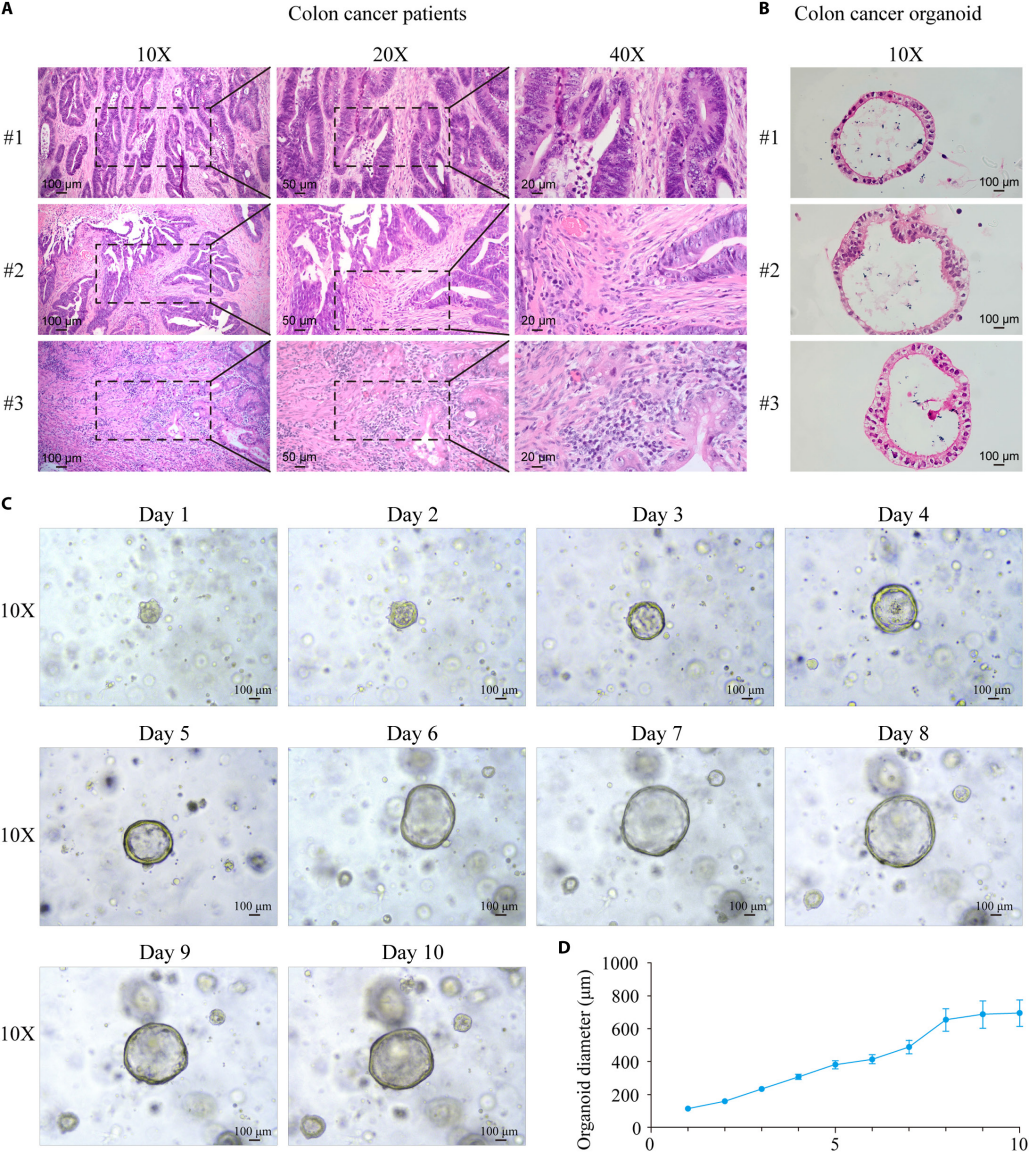
Tissue resources and organoid construction of colon cancer. (A) H&E staining of surgically resected tissues of 3 patients with colon cancer. (B) H&E staining of organoids of colon cancer. (C) Dynamic growth status of colon cancer organoids. (D) Growth curve of colon cancer organoids.

With the informed consent of the patients, postoperative tissue samples were collected from the Department of General Surgery and were confirmed as colorectal adenocarcinoma by pathological examination (Fig. 3A). The tissue samples were collected and kept at 4 °C to maintain cell activity and stability. Fat and muscle tissues were carefully removed and cut into small tissue fragments of about 1 mm to prepare single-cell suspension of tumor tissues for the construction and culture of colon cancer organoids. Hematoxylin and eosin (H&E) staining verified the consistency of organoid histology with colon cancer (Fig. 3B). According to the growth status (Fig. 3C) and growth curve (Fig. 3D) of the organoids, the organoids were taken out for experiment when the diameters were about 600 μm.

### Neutrophil micro-injection could significantly decrease organoid diameters

**Fig. 4.**
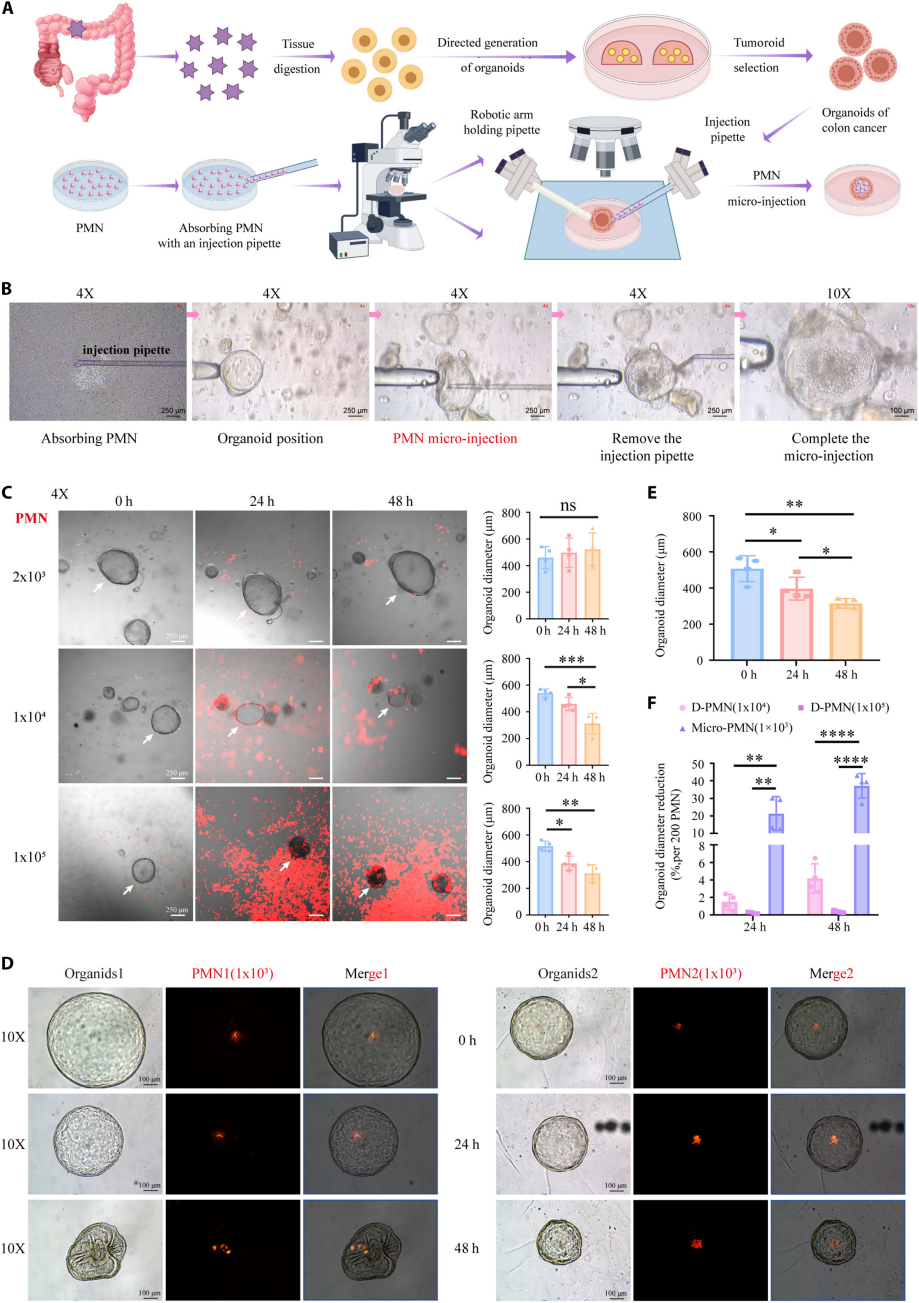
Colon cancer organoid neutrophil micro-injection. (A) Pattern of colon cancer organoid construction and neutrophil micro-injection. (B) Process of neutrophil micro-injection under microscope. (C) Direct coculture of neutrophils and colon cancer organoids. (D) Neutrophil micro-injection of colon cancer organoids. (E) Changes in diameters of organoids by neutrophil micro-injection. (F) Percentage reduction in colon cancer organoid diameter by per unit number of neutrophils (1 × 10^3^). D-PMN, directly coculture of neutrophils and organoids; Micro-PMN, neutrophil micro-injection of organoids. ns, no significance. *P < 0.05, **P < 0.01, ***P < 0.001 compared to the control group. PMN, polymorphonuclear, another term for a neutrophil.

The process of organoid construction and neutrophil micro-injection for colon cancer is shown in Fig. 4A. The neutrophil micro-injection consists of 4 steps: neutrophils aspirated by injection pipette, fixation of organoids, micro-injection of neutrophils, and withdrawal of injection pipette (Fig. 4B and Movie S2). Colon cancer organoids were directly cocultured with neutrophils (2 × 10^3^, 1 × 10^4^, and 1 × 10^5^), and the results manifested that colon cancer organoids did not show significant effects when cocultured with the minimum amount of neutrophils (2 × 10^3^), but the diameters of colon cancer organoids were decreased in 48 h by increasing the number of neutrophils (1 × 10^4^ and 1 × 10^5^) (Fig. 4C). Moreover, it is noteworthy that the diameters of colon cancer organoids were reduced by micro-injection of 1 × 10^3^ neutrophils at 24 h, and the tissue shrinkage and diameter reduction became more distinct at 48 h (Fig. 4D and E). By calculating the percentage of diameter reduction of colon cancer organoids caused by unit number (1 × 10^3^) of neutrophils, it was found that neutrophil infiltration presented a more apparent inhibitory effect on growth of colon cancer organoids (Fig. 4F). It proved that a sufficient intratumoral number of neutrophils could play an effective antitumor role.

### 3D-printed coculture model revealed neutrophil-mediated killing of colon cancer cells

**Fig. 5.**
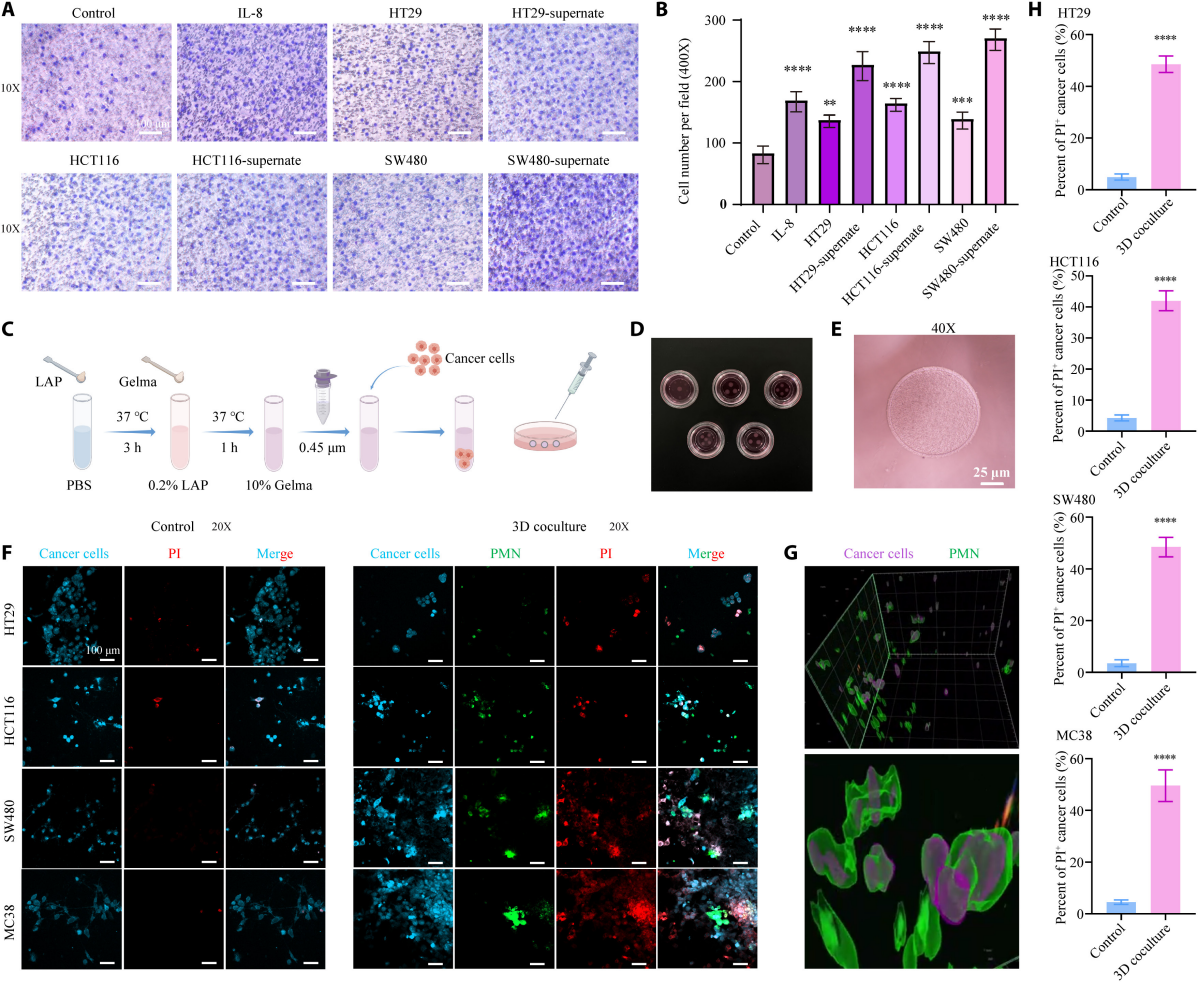
3D-printed coculture model revealing neutrophil killing of colon cancer cells. (A) Chemotaxis of human neutrophils under different conditions. (B) Number of neutrophil chemotactic cells under different conditions. (C) Construction of the 3D-printed coculture model. (D) Naked-eye images of the 3D-printed coculture model. (E) Microscopic images of the 3D-printed coculture model. (F) Fluorescence images of the cell death state of the tumor cells in 2 states, namely, the natural growth state and coculture with neutrophils. (G) Spatial positional relationship between the neutrophils and the tumor cells within the 3D-printed coculture model. (H) Quantitative analysis of dead tumor cell proportions before and after co-culture with neutrophils. Scale bar, 100 μm. **P < 0.01, ***P < 0.001, ****P < 0.0001 compared to the control group.

Transwell cell chemotaxis assay confirmed that human neutrophils exhibited significant chemotaxis toward IL-8, colon cancer cells, and cancer cell culture supernatants (Fig. 5A). The number of chemotactic cells was significantly higher compared to the control group (Fig. 5B). To stimulate a 3D tumor microenvironment (Fig. 5C to E), Gelma wrapping of tumor cells was used to observe neutrophil infiltration and activity within the microenvironment, thus mimicking the in vivo state. Tumor cells are prestained with a membrane tracer before wrapping, allowing the dye to pass on as cells divide and proliferate, facilitating the observation of tumor cells. However, neutrophils were differentially labeled with a tracer of another color. After 24 h of coculture, dead cells were stained using propidium iodide (PI) dye. Under normal culture conditions, the tumor cells exhibited good growth with few PI-positive cells. In contrast, the coculture group exhibited a large number of PI-positive cells, with most colocalizing with tumor cells (Fig. 5F and H and Fig. S5). This indicates that neutrophils entered into the tumor microenvironment and killed tumor cells. The model was imaged in 3D using confocal microscopy, eliminating the effect of stray color. Neutrophils entered the tumor microenvironment and formed clusters, encapsulating the tumor cells at the center (Fig. 5G and Movie S3), consistent with results from the aforementioned microscopy analyses.

### Neutrophils induce apoptosis in colon cancer cells by generating NETs and releasing NE

**Fig. 6.**
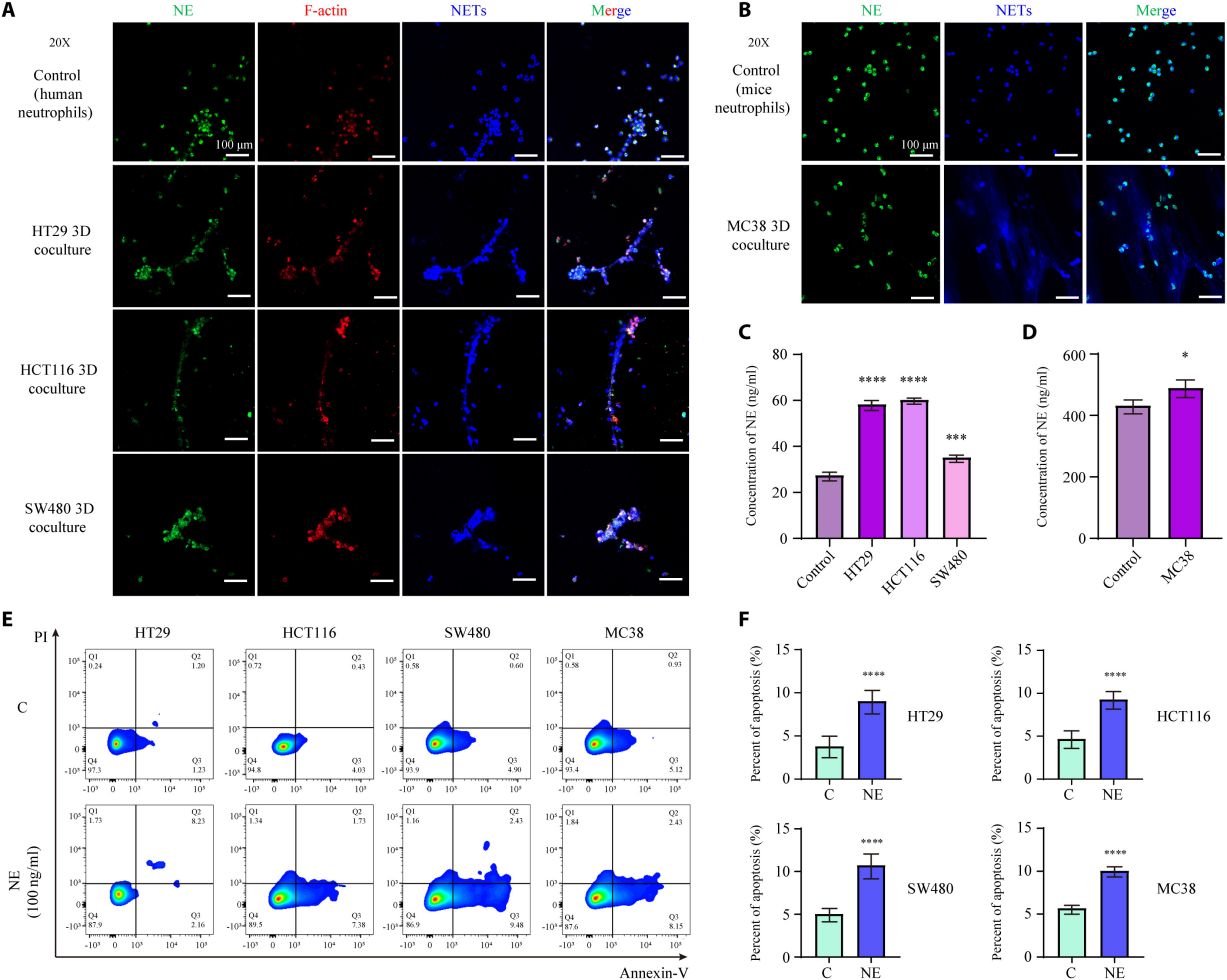
NETs and NE releasing promote apoptosis of tumor cells. (A) Fluorescence images of human neutrophils under normal culture conditions and coculture with HT29, HCT116, and SW480 cells. (B) Fluorescence images of mouse neutrophils under normal culture conditions and coculture with MC38 cells. (C) Comparison of NE content in the cell supernatants of human neutrophils under normal culture conditions and coculture with various colon cancer cells in both states. (D) Comparison of NE content in cell supernatants of mouse neutrophils under normal culture conditions and coculture with MC38 colon cancer cells in 2 states. (E) Apoptosis profiles of various colon cancer cells after 24 h of NE (100 ng/ml) intervention. (F) Apoptosis rate analysis of HT29, HCT116, SW480, and MC38 cells after 24 h of NE intervention. Scale bar, 100 μm. *P < 0.05, ***P < 0.001, ****P < 0.0001 compared to the control group.

Staining for the cytoskeleton (red), NETs (blue), and NE (green), the cocultured neutrophils generated extensive NETs and exhibited a disorganized and unclear cytoskeleton. In control cells, most NE proteins were concentrated intracellularly, whereas in cocultured cells, NE proteins were primarily located at the membrane, with vacancies observed in the cytoplasm (Fig. 6A and B). It was hypothesized that NE proteins transitioned from intracellular to extracellular regions as NETs were released. To verify this, NE protein levels in the supernatant of each group were measured. The concentration of NE protein in the supernatant of each coculture group was significantly higher than that of the control group (Fig. 6C and D). Tumor cells were stimulated and cultured with purified human- and mouse-derived NE protein (100 ng/ml). The results indicated that NE protein could indeed induce apoptosis in tumor cells (Fig. 6E). Furthermore, the apoptosis rate in each NE intervention group was significantly higher than that in the control group (Fig. 6F).

### Bone marrow-derived neutrophils present complete mature neutrophil functionality in mice

**Fig. 7.**
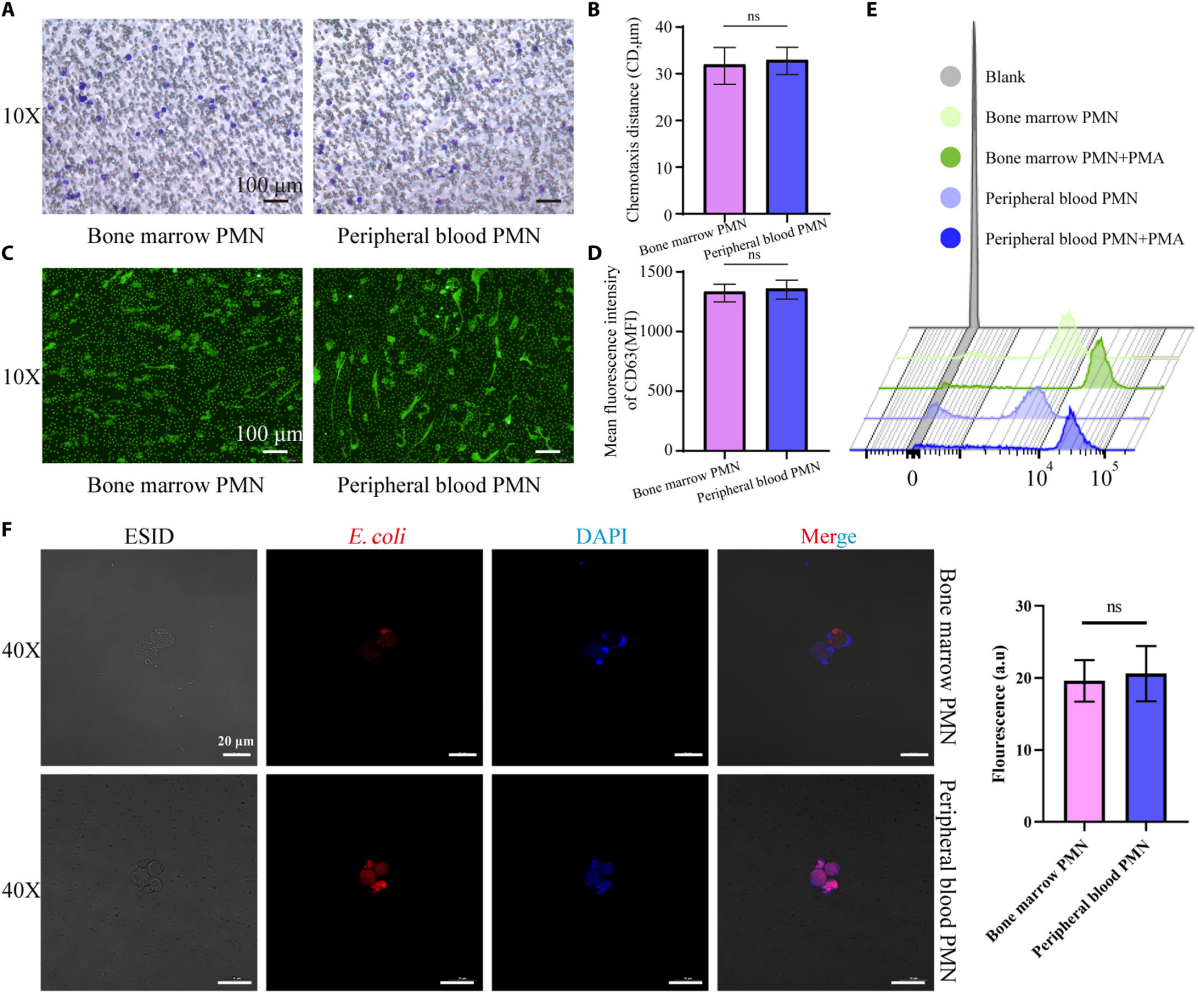
Neutrophils in bone marrow possess the same biological function as neutrophils in peripheral blood in mice. Neutrophils in bone marrow and peripheral blood possess identical chemotactic function (A, scale bar, 100 μm; B, no significance in neutrophil chemotaxis distance), NE formation (C, scale bar, 100 μm), degranulation (D), oxygen burst capacity (E), and phagocytosis function (F, scale bar, 20 μm). There were 3 mice in each group, and the experiments were repeated 3 times.

Transwell assay revealed that neutrophils derived from bone marrow have the same chemotactic function with those from peripheral blood in mice (Fig. 7A and B). Similarly, bone marrow-derived neutrophils could generate NETs after phorbol 12-myristate 13-acetate (PMA) stimulation as neutrophils in peripheral blood (Fig. 7C), and there is no difference in degranulation function (expression of azurophilic granule CD63) (Fig. 7D) and respiratory oxygen burst ability (Fig. 7E) between them. What is more, they also have intact phagocytic function (Fig. 7F). These results highlighted the intact functionality of bone marrow-derived neutrophils, making them suitable for allogeneic transfusion.

### NAT is safe

**Fig. 8.**
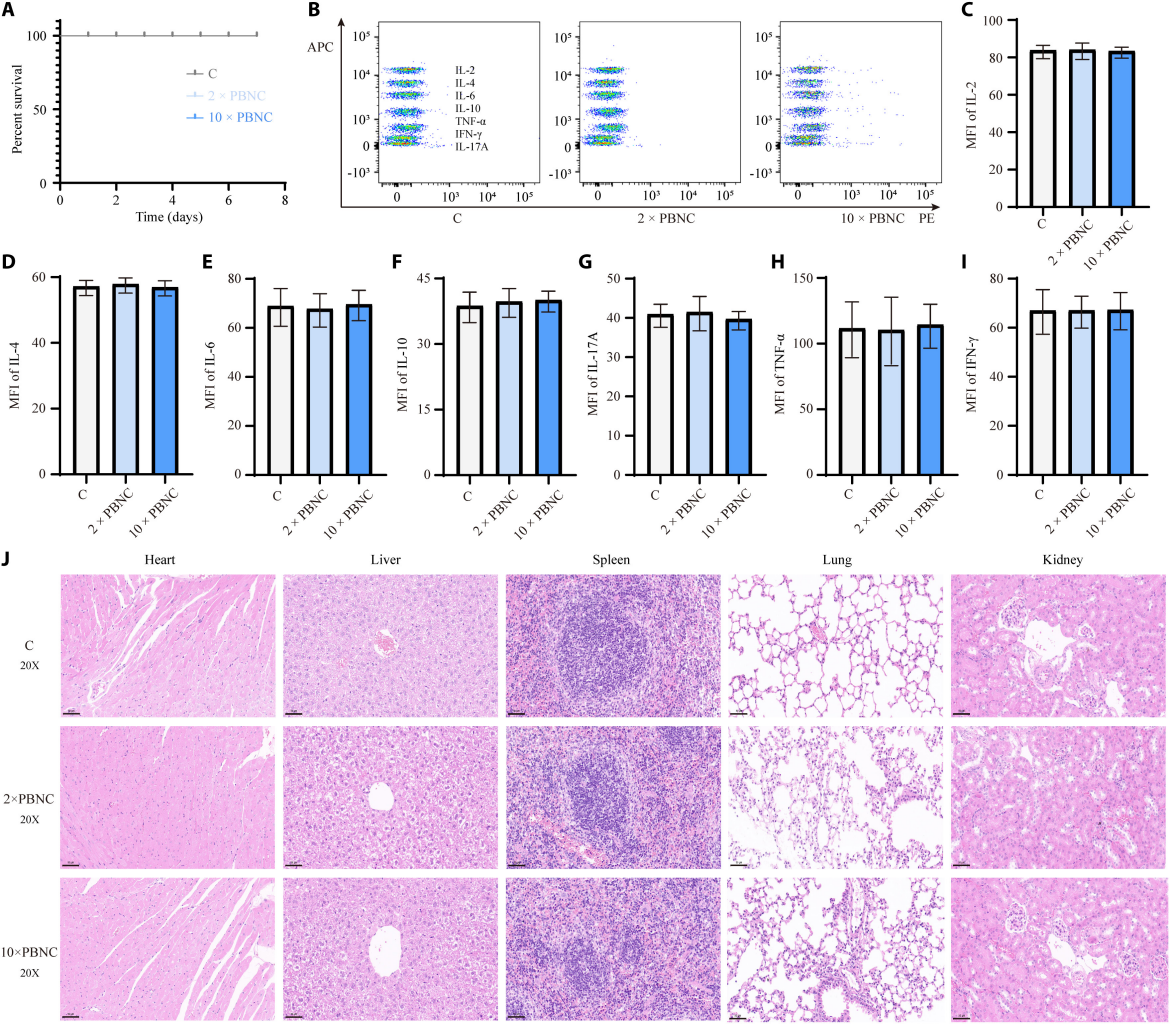
Safety evaluation of allogeneic neutrophil transfusion. (A) Survival rate statistics of mice in the control, 2×PBNC, and 10×PBNC groups. (B) Plasma inflammatory factor levels in the 3 groups of mice. Statistical analysis of plasma IL-2 (C), IL-4 (D), IL-6 (E), IL-10 (F), IL-17A (G), TNF-α (H), and INF-γ (I) contents in the 3 groups of mice. (J) Observation of H&E sections of organs from the 3 groups of mice. Scale bar, 50 μm. There were 3 mice in each group, and the experiments were repeated 3 times.

The neutrophils are abundant in bone marrow and possess the same biological function as neutrophils in peripheral blood in mice. The safety of allogeneic transfusion was assessed using doses equivalent to 2 and 10 times the number of circulating neutrophils in the peripheral blood of mice. Post-transfusion, the mice exhibited good mental status, normal daily behavioral activities, stable appetite, and body weight. Additionally, no mortality occurred in any of the experimental groups (Fig. 8A). The plasma inflammation-related indexes, including IL-2, IL-4, IL-6, IL-10, IL-17A, tumor necrosis factor-α (TNF-α), and interferon-γ (INF-γ), were measured 6 h after NAT (Fig. 8B to I). The results suggested no significant changes in these inflammatory markers. Histological examination of H&E-stained sections from several organs, including the heart, liver, spleen, lung, and kidney, revealed no significant differences or signs of organ damage across all groups (Fig. 8J). Based on these findings, 2-fold peripheral blood neutrophil counts (PBNCs) were determined and used as the sufficient cell count for allogeneic transfusion in subsequent experiments.

### Neutrophils are essential inhibitory factors for tumor growth

**Fig. 9.**
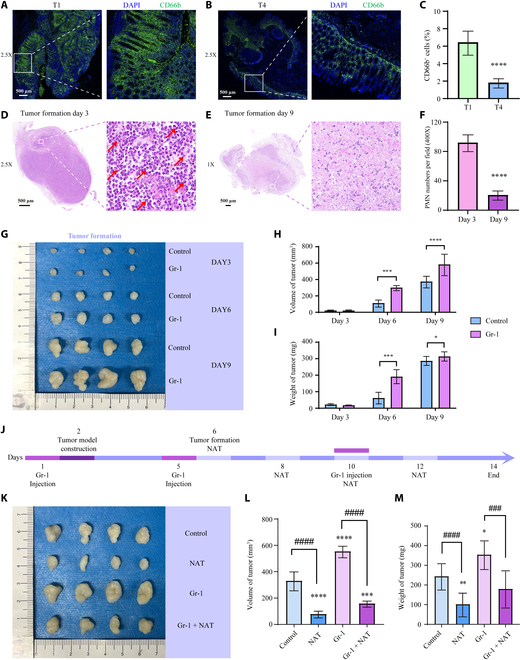
Role of neutrophils in colon cancer tumorigenesis. (A) Neutrophil infiltration in the colon of patients with stage T1 and (B) T4 colon cancer. (C) Analysis of the percentage of infiltrating neutrophils in patients with stage T1 and T4. Intratumor neutrophil infiltration on days 3 (D) and 9 (E) of subcutaneous tumorigenesis in mice. (F) Comparison of the number of intratumor neutrophil infiltration on days 3 and 9 of subcutaneous tumorigenesis in mice. (G) Dynamic tumorigenic status of control mice and neutrophil-cleared mice compared with the dynamic tumorigenic volume (H) and weight (I). (J) Construction of the tumor model of NAT mice. (K) Tumorigenic results of the 4 groups of mice: control group, NAT group, Gr-1 group, and Gr-1 + NAT group. Comparison of the tumorigenic volume (L) and weight (M) of the 4 groups of mice. Scale bar, 500 μm. *P < 0.05, ***P < 0.001, ****P < 0.0001 compared to the control group. ANOVA for comparison among multiple groups, ^###^*P* < 0.001, ^####^*P* < 0.0001. There were 4 mice in each group, and the experiments were repeated 3 times.

Surgically resected colon specimens from patients with stage T1 and T4 colon cancer were stained to assess neutrophil infiltration. Neutrophils were labeled using CD66b fluorescent primary antibody, and all cell nuclei were stained with 4′,6-diamidino-2-phenylindole (DAPI) (Fig. 9A and B). Then, the percentage of CD66b^+^ cells relative to total cells was significantly higher in stage T1 patients than in stage T4 patients (Fig. 9C). In animal experiments, H&E staining was performed on colon cancer samples from mice on days 3 and 9 of tumorigenesis (Fig. 9D and E). Subsequently, the number of polymorphonuclear cells in the field of view was counted, revealing a significantly higher intratumoral neutrophil count on day 3 than on day 9 of tumorigenesis (Fig. 9F). The same results were corroborated in flow cytometry analysis of tumor tissue-derived single-cell suspensions, which demonstrated a comparable proportion of neutrophils (Fig. S6), indicating the important role of neutrophils in early tumorigenesis in mice. Therefore, neutrophils were depleted in vivo using the Gr-1 antibody, which was replenished every 5 d to maintain the neutrophil depletion. A neutrophil-cleared mouse model was constructed, and subcutaneous tumor inoculation was performed to dynamically observe tumor growth. Significant differences in tumor growth were observed between control mice (control group) and neutrophil-cleared (Gr-1 group) mice on days 6 and 9 of tumorigenesis. Additionally, the volume and weight of the tumors in the Gr-1 group were significantly higher than those in the control group (Fig. 9G to I). Additionally, the aggressive dose of fresh neutrophil NAT mouse model was constructed. NAT was administered every 2 d post-tumorigenesis. The experiment was uniformly concluded on day 9 of tumorigenesis in mice. The specific experimental procedure is shown in Fig. 9J. Comparisons between NAT or not (control versus NAT, Gr-1 versus Gr-1 + NAT) showed that tumor volume and weight were significantly smaller in NAT mice. Consistent with dynamic tumorigenesis assay results, mice in the Gr-1 group had significantly higher tumor volume and weight than those in the control group (Fig. 9K to M).

### IL-8 combined with NAT can generate a sufficient number of neutrophils in the tumor microenvironment

**Fig. 10.**
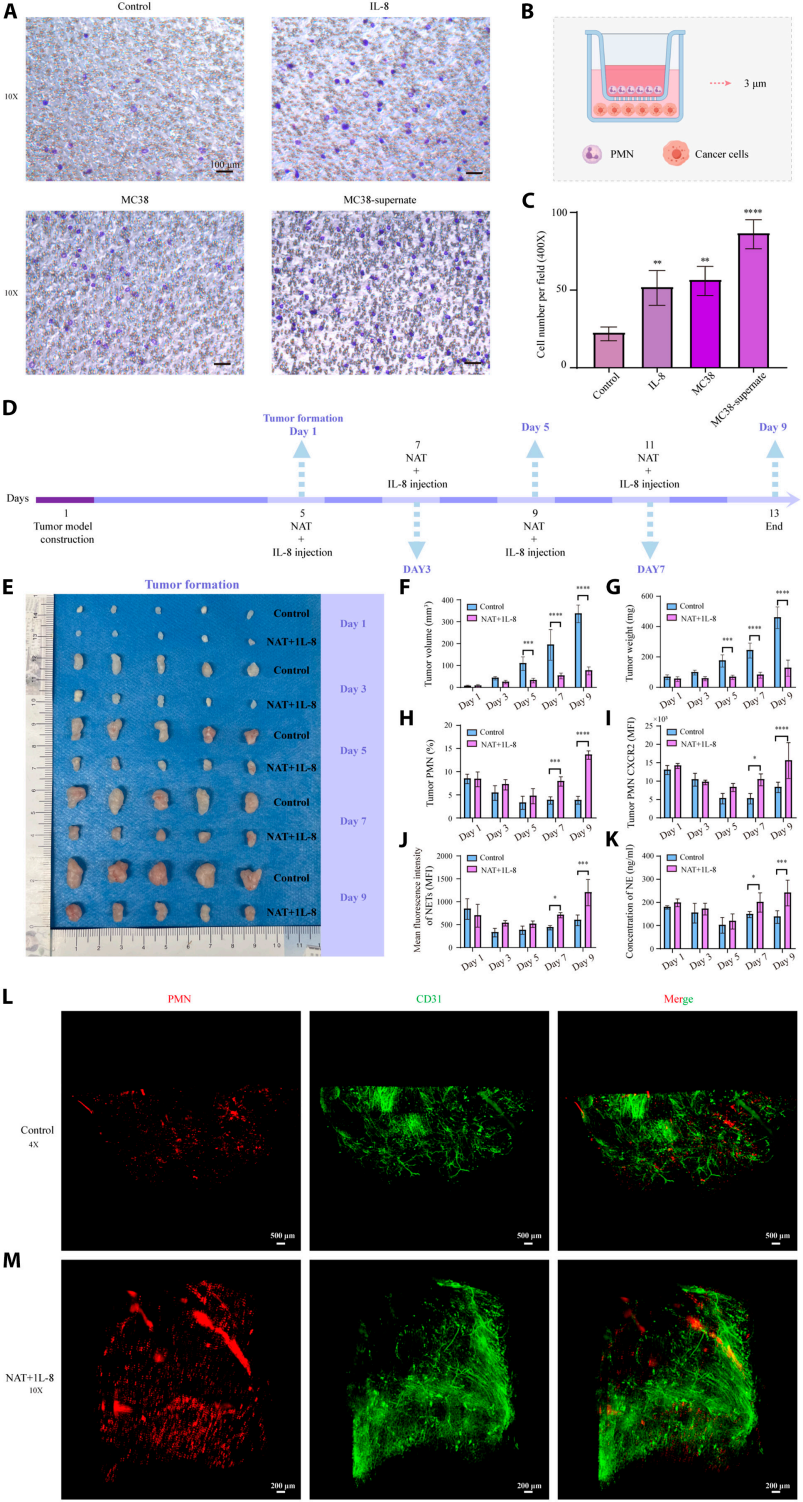
Mouse subcutaneous tumorigenesis suppressed by IL-8 combined with NAT. (A) Chemotaxis images of mouse neutrophils under different conditions. Scale bar, 100 μm. (B) Schematic representation of the Transwell (3 μm) chemotaxis assay for detecting mouse neutrophil chemotaxis. (C) Analysis of the number of chemotactic neutrophils under different conditions. (D) Mouse tumor model constructed by NAT combined with peritumoral injection of IL-8. (E) Dynamic tumorigenesis results of control mice and treated mice. Comparison of the dynamic tumorigenic volume (F), weight (G), the percentage of intratumoral neutrophils (H), the expression of intratumoral neutrophil CXCR2 (I), the number of NETs generated (J), and the NE content (K) in the tumor tissue grinding solution in both groups of mice. 3D image of tumor hyalinization treatment after tumorigenesis in (L) control mice (scale bar, 500 μm) and (M) NAT + IL-8 group mice (scale bar, 200 μm). *P < 0.05, **P < 0.01, ***P < 0.001, ****P < 0.0001 compared to the control group. There were 5 mice in each group, and the experiments were repeated 3 times.

IL-8 is a common inflammatory factor secreted by tumor cells. The chemotaxis function of neutrophils was verified using a Transwell cell chemotaxis assay (Fig. 10B). Supernatant culture solutions from the control [1640 medium containing 10% fetal bovine serum (FBS)], IL-8 (500 ng/ml), MC38 cells (2 × 10^5^ cells), and MC38 cells after 24 h of culture were collected. Neutrophils showed significant chemotaxis toward IL-8, MC38 cells, and MC38 cell culture supernatants (Fig. 10A), with a significantly increased number of chemotactic cells compared to the control group (Fig. 10C). Subsequently, NAT was administered to tumor-bearing mice, followed by peritumoral injection of IL-8 (500 ng/ml, 100 μl/each). The infused fresh neutrophils were attracted to the tumor site. The experimental procedure is shown in Fig. 10D. Peritumoral subcutaneous injection of IL-8 did not significantly inhibit the growth of tumors (Fig. S7). Therefore, the biological effects of IL-8 itself could be considered negligible under these specific intervention conditions. From day 5 of tumorigenesis, the treated group (later referred to as the NAT + IL-8 groups) exhibited significantly lower tumor volume and weight compared to the untreated group (Fig. 10F and G). By day 7 of tumorigenesis, the tumors of untreated mice tended to invade the muscles and the treated group showed higher percentages of neutrophils in the tumor (Fig. 10H), increased expression of neutrophil CXCR2 (Fig. 10I), enhanced production of NETs (Fig. 10J), and elevated concentration of NE protein in the tumor tissue grinding fluid (Fig. 10K) compared to the untreated group (Fig. 10H and I). Additionally, on day 9 of tumorigenesis, mice in both the control and treated groups underwent cardiac perfusion, and tumors were isolated for tissue hyalinization. Furthermore, neutrophils and blood vessels were stained, and 3D imaging was performed to examine neutrophil distribution within the tumors of both groups (Movie S4). The imaging revealed a significant increase in neutrophils within the tumors of mice in the treatment group (Fig. 10L). Moreover, while the control group exhibited a large tumor volume and the presence of neutrophils within the tumor, their distribution was scattered, contrasting with the treatment group (Fig. 10M). These results suggest that neutrophils are attracted to IL-8 for chemotaxis in colon cancer mice and NAT can significantly inhibit tumor growth.

## Discussion

Neutrophils, the most abundant type of leukocytes in the human peripheral circulatory system, play crucial roles in tumor growth, metastasis, and immune evasion [34,35]. Many studies have confirmed that neutrophils can accumulate at various tumor sites [36,37]. Within the tumor microenvironment, TANs can exert both protumor and antitumor effects, making it challenging to define them unequivocally as “friend” or “foe” [38].

**Fig. 11.**
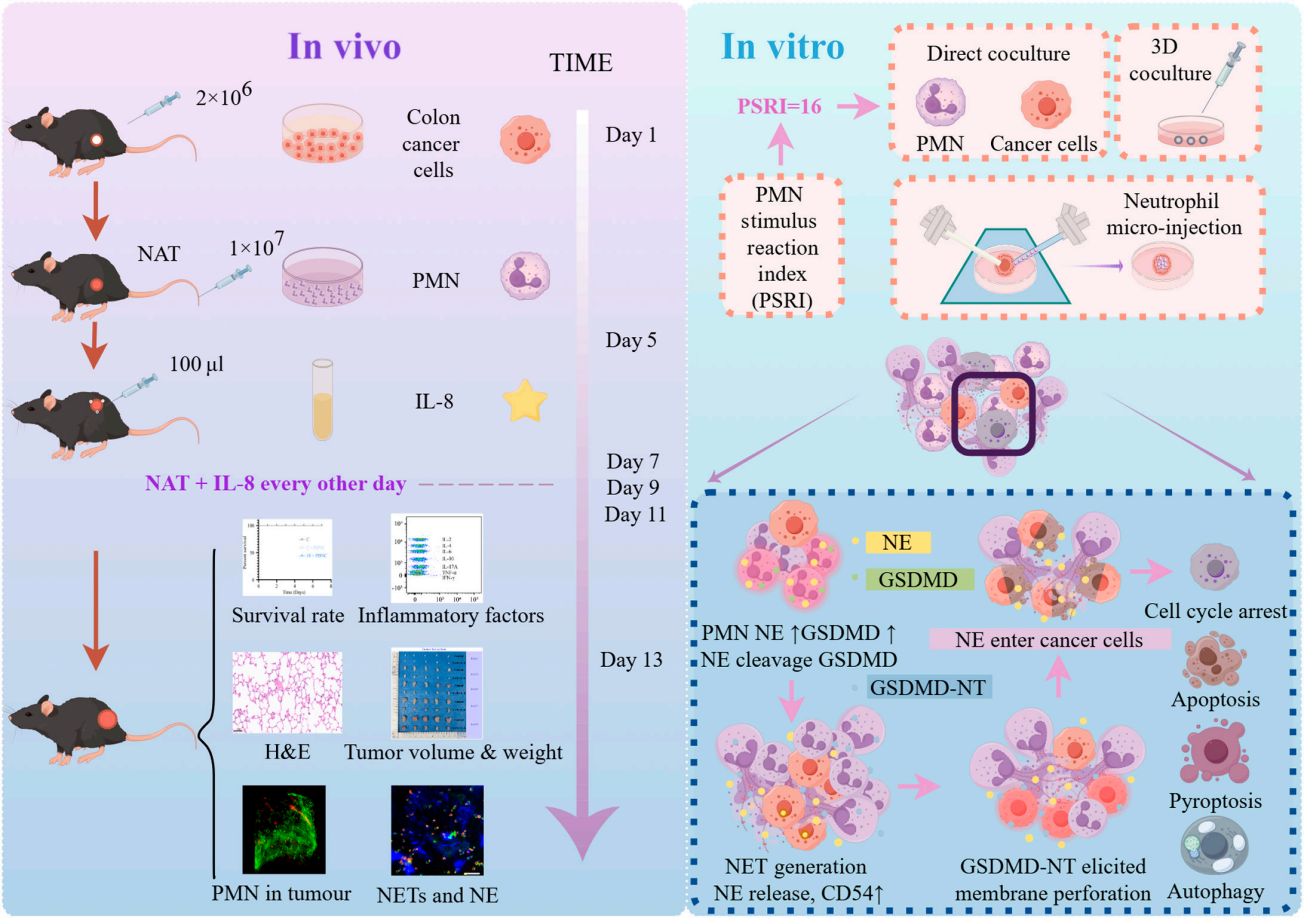
Schematic representation of an aggressive dose fresh neutrophil-mediated killing effects for colon cancer. In vitro coculture experiments, platform of colon cancer organoid neutrophil micro-injection, 3D-printed tumor microenvironment models, and in vivo tumor-bearing mice NAT treatment experiments revealed that neutrophils, NETs, and NE in the tumor microenvironment act synergistically and exert their antitumor effects with high efficiency.

While neutrophils have traditionally been recognized for their anti-inflammatory properties, their functional dynamics within tumor microenvironments, particularly the pleiotropic nature of TANs, remain poorly characterized. This incomplete understanding of neutrophil biology in oncogenesis, coupled with uncertainties regarding their antitumor mechanisms, has significantly constrained the development of neutrophil-based therapeutic approaches. To address these limitations, NSRI was preliminary established to systematically identify optimal neutrophil donors, thereby ensuring the isolation of functionally intact neutrophils under stringent selection criteria. Leveraging this platform, we aimed to elucidate the role played by fresh neutrophils with precise navigation in the development of colon cancer (Fig. 11). The results indicated that a sufficient infiltration of functionally competent neutrophils can markedly inhibit tumor growth. Analyses revealed that direct neutrophil–tumor cell contact mediates potent cytotoxic activity, manifesting as significant cell cycle arrest and markedly enhanced apoptotic indices in malignant cells. In the tumor microenvironment, neutrophil activation triggers enhanced production of intracellular granules and antimicrobial proteins, including neutrophil elastase (NE). This protease cleaves GSDMD to generate its active N-terminal fragment (GSDMD-NT), which forms pores in plasma membranes to facilitate NETs formation. NETs represent a unique neutrophil effector mechanism, comprising decondensed chromatin decorated with antimicrobial proteins released during neutrophil activation [39]. They were originally characterized for roles in capturing and eliminating microbial pathogens through the high local concentrations of bactericidal proteins, establishing them as a vital extracellular immune defense mechanism [40–42]. Our findings reveal that NETs mediate extracellular release of both NE and GSDMD-NT. The latter perforates colon cancer cell membranes, enabling NE entry and subsequent intracellular damage, ultimately inducing multimodal cell death with predominant apoptotic features. This represents a previously unrecognized antitumor mechanism of neutrophil-mediated cytotoxicity. Furthermore, we systematically analyzed the effects of different count of neutrophils on growth of organoid colon cancer in the platform of colon cancer organoids with neutrophil micro-injection for the first time. As expected, neutrophils inhibited the growth of colon cancer organoids in a concentration-dependent manner. In terms of the size, shape, and percentage reduction in diameters of organoids, the results showed that organoid growth was significantly suppressed after micro-injection of neutrophils, which indicated that a small number of neutrophils infiltrating into the tumor can also effectively exert their antitumor ability. These also verified the tumor-killing ability of neutrophils from another aspect. Therefore, how to induce neutrophil infiltration in the early stage of tumor is a very important proposition. Similar results were observed using a 3D-printed coculture model to simulate the tumor microenvironment. Neutrophils possess strong chemotactic abilities, allowing them to respond swiftly to inflammatory signals from the tumor site, infiltrating the tumor microenvironment to kill cancer cells. In the 3D-printed coculture model, most colon cancer cells died, while neutrophils underwent cytoskeletal rearrangement, generating large quantities of NETs and releasing intracellular NE into the extracellular environment. In vitro experiments confirmed the proapoptotic effects of NE on tumor cells. Longitudinal monitoring of tumor growth dynamics demonstrated that neutrophil-depleted mice exhibited significantly greater tumor volumes and mass compared to control animals from day 6 of tumorigenesis. These findings establish that NAT employing fresh, functionally competent neutrophils can effectively suppress tumor progression. Integrating in vivo and in vitro investigations consistently demonstrated that neutrophils induce apoptosis in colon cancer cells through a NET-dependent mechanism involving NE release. The antitumor mechanism of NE has been universally acknowledged. It has been demonstrated that ELANE proteolysis releases the CD95 death domain, which interacts with the histone H1 subtype to selectively kill tumor cells. NE also inhibited the growth of primary tumors and produced a distant effect mediated by CD8^+^ T cells to attack distant metastases [43,44]. Therefore, our study has not conducted in-depth research on specific mechanism of NE. Moreover, although NETs have been implicated in promoting metastatic dissemination in certain contexts [45], in this study, no metastasis was found in tumor-bearing mice, and we consider that only NETs produced by sufficient number of fresh PMN can perform the tumor-killing effect.

Tumor cells secrete multiple inflammatory chemokines, primarily mediating neutrophil migration to tumors via CXC chemokines such as CXCR1 and/or CXCR2 [46,47]. IL-8, a predominant cytokine produced by tumor cells, prompts neutrophils to rapidly respond to IL-8 calls and exhibit chemotaxis to the target site. Further validation of the number and biosafety of NAT identified a 2-fold neutrophil transfusion in peripheral blood as the aggressive intervention dose. Additionally, peritumoral IL-8 injection combined with NAT induced the formation of a sufficient intratumoral number of neutrophils and promoted neutrophil wrapping around the tumor. IL-8-mediated navigational mode therapy can enhance the local role of neutrophils and reduce systemic inflammatory damage. From day 5 of tumorigeneses, the volume and weight of tumors in mice receiving this combination treatment were significantly lower than in control mice. Moreover, from day 7 onward, the percentage of neutrophils in the tumor and the expression of CXCR2, a receptor for IL-8 [48,49], were significantly higher in the treatment group. This suggests enhanced neutrophil response to IL-8 and increased chemotactic ability, correlating with the higher percentage of neutrophils in the tumors of the treatment group. Dynamic tracking of tumor growth revealed day 5 of tumorigenesis as the critical point where significant differences between the 2 groups emerged, coinciding with the completion of 3 treatment cycles. This suggests a complex entry pattern of neutrophils into tumors, with variable entry rates and dynamic behavior changes post-entry. Day 5 marks the initiation of the tumor-killing effect by neutrophils, triggering a cascade of immune responses within the tumor microenvironment, contributing to antitumor effects in conjunction with other immune cells. Consequently, significant differences in intratumor neutrophil numbers were observed on days 7 and 9 of tumorigenesis. These findings underscore the importance of maintaining a sufficient intratumor number of neutrophils. NAT therapy, therefore, requires a continuous course of treatment. A single transfusion of neutrophils may be counterproductive due to the tumor microenvironment’s potential to subvert the neutrophils, promoting tumor growth. However, intermittent sequential therapy can effectively harness neutrophils’ antitumor potential.

Nonetheless, exogenous neutrophils represent allogeneic cells to recipients, and their transfusion may pose potential safety risks, including the induction of immune rejection reactions in hosts. Studies suggest that these reactions may be associated with human leukocyte antigen (HLA) expression on neutrophil surfaces, as well as residual red blood cells and monocytes in the preparations [50]. To mitigate immunogenicity, consideration should be given to using umbilical cord blood-derived neutrophils and implementing additional purification steps to minimize erythrocyte contamination [51].

Clinical observations indicate that febrile reactions are common following neutrophil transfusion, although the temperature elevation typically remains within an acceptable range. Current evidence suggests that fever may be more related to recipient factors than the transfusion itself [52]. Regarding transfusion-related acute lung injury (TRALI), insufficient evidence exists to determine whether differences in pulmonary adverse events occur between recipients who receive therapeutic granulocyte transfusions and those who do not [53]. The occurrence of cytokine storm reactions in a subset of recipients underscores the necessity of prophylactic antibiotic and antifungal therapy [54,55]. Therefore, patients receiving neutrophil transfusion therapy require rigorous vital sign monitoring to promptly address any potential severe adverse reactions.

Cancer, a highly complex disease, challenges the immune system, which serves as a natural barrier against foreign pathogens and maintains human health. Effective tumor treatment leverages the immune system’s “internal force” alongside the “external force” of other interventions to destroy tumor cells. Neutrophils, key players in the tumor immune response, show increased subpopulations within the tumor microenvironment and exhibit substantial intermediate states among various populations [56,57]. Specific tumor-infiltrating neutrophils can exert antitumor effects [58,59]. The tumor microenvironment is a complex ecosystem where immune system suppression plays a pivotal role in tumorigenesis and progression [15,60]. It keeps antitumor immune cells in an activated “alert” state, overcoming tumor immune escape, and dismantling the tumor from within, thus enhancing the effectiveness of immunotherapy. This study elucidates a novel neutrophil-mediated cytotoxic mechanism through GSDMD–NT–neutrophil extracellular traps (NETs)–NE and demonstrates that combining NAT with peritumoral IL-8 injection effectively inhibits tumor growth, presenting a novel and promising strategy for tumor therapy—“highly selectivity and precise navigation neutrophil therapy”. The further correlative preclinical studies to expedite the clinical application of neutrophil-directed therapeutic regimens will be proceeded. Additionally, novel techniques that induce noninvasive inflammation through local low-dose radiotherapy in tumors, leading to “self-navigation” neutrophil aggregation, are under exploration. Neutrophil navigational therapy opens new avenues for tumor immunotherapy and has optimistic clinical application prospect.

## Conclusion

Allogeneic transfusion of an aggressive dose fresh neutrophils with precise navigation can stimulate neutrophil infiltration within the tumor microenvironment and induce multimodal death in colon cancer cells by generating NETs and releasing GSDMD-NT and NE. Neutrophil navigational therapy paves the way for advancements in neutrophil-based immunotherapy for cancer treatment.

## Materials and Methods

### Purification of fresh human and mouse neutrophils

Human peripheral blood and mouse bone marrow neutrophils were purified using the EasySep Direct Human Neutrophil Isolation Kit (STEMCELL, 19666) and Mouse Neutrophil Negative Selection Kit (STEMCELL, 19761), respectively. Noteworthily, the process of purified cells should avert vibrating, temperature variation, and rough operation to avoid neutrophil activation. The purified neutrophils do not undergo any treatment (including activation and function training) and be used within 1 h to to ensure freshness and viability of them.

### Establishment of NSRI for high selectivity of neutrophils

NSRI was composed of 4 indicators: P2X1 (a chemotaxis indicator) [61,62], CD10 (a maturity indicator) [63,64], CD66b-LPS/C (a degranulation indicator; LPS/C, the ratio of expression of neutrophils after LPS stimulation to that untreated control cells ) [65,66], and CD11b-LPS/C (an activation indicator) [67,68] of neutrophils (Table). We recruited 220 healthy volunteers from the Suzhou Medical Examination Center to establish a normal range for the NSRI. The data distribution for these 4 indicators was shown in Fig. S1, all of which are non-normally distributed. Setting the 95% confidence interval, the normal ranges are as follows: P2X1 ≤ 437, CD10 ≥ 2,622, CD66b (LPS/C) ≥ 1.11, and CD11b (LPS/C) ≥ 1.07. Based on the quartiles and medians of each indicator, we determined that a score of 16 is normal for neutrophil stimulus response. Scores of 12 to 15, 8 to 11, 5 to 7, and ≤4 are classified as mild, moderate, severe, and extremely severe abnormalities in the NSRI, respectively. In each experiment, 3 healthy volunteers were selected to evaluate the NSRI of their peripheral blood neutrophils, and cells from volunteers who scored 16 were chosen for further experimentation. In animal experiments, 3 mice were selected for each experiment. In the neutrophil chemotaxis experiment toward IL-8 using a Transwell system, only when the number of cells per field reached 100 and the expression of CD63 in neutrophils was higher than 200, the cells were selected for further studies.

**Table.** Scoring table of NSRI.

Neutrophil stimulus reaction index (PSRI)						
Indicators	Scoring standards					
	0	1	2	3	4	Score
P2X1	≥437	284–437	233–284	167–233	<167	
CD10	≤2,622	2,622–4,018.5	4,018.5–5,034	5,034–6,069	>6,069	
CD66b (LPS/C)	≤1.11	1.11–1.44	1.44–1.71	1.71–2.06	>2.06	
CD11b (LPS/C)	≤1.07	1.07–1.26	1.26–1.45	1.45–1.68	>1.68	
Neutrophil stimulus reaction				Score		
Normal				16		
Abnormal		Mild		12–15		
		Moderate		8–11		
		Severe		5–7		
		Extremely		≤4		

### Cell cycle and apoptosis detection

Digested cells were stained with neutrophil marker flow antibodies (human, CD66b; mouse, Ly6G) for 30 min at room temperature. Subsequently, the samples were incubated with apoptosis flow antibodies (BD, 556420, each group of cells was stained with 100 μl of buffer + 5 μl of Annexin-V + 5 μl of PI) for 15 min at room temperature. Cell cycle was detected by Cell Cycle Assay Kit-PI/RNase Staining (Dojindo, C543). Each group of cells was stained with 500 μl of assay buffer + 25 μl of PI solution + 2.5 μl of ribonuclease (RNase) solution for 30 min at 37 °C. Then, 400 μl of buffer was added to detect apoptosis and cell cycle utilizing a flow cytometry.

### Construction and culture of colon cancer organoids

The surgically isolated human colorectal cancer tissues were stored in tissue storage solution (BioGenous, K601005) and transported at 4 °C. The fat and muscle tissue were carefully removed with surgical scissors. The tissue samples were repeatedly cleaned with ice phosphate-buffered saline (PBS) and cut to small tissue fragments of about 1 mm in diameter. Tumor tissues were digested with tumor tissue digestible solution (BioGenous, K601003) for 30 min at 37 °C, mixed every 5 min. FBS (final concentration, 2%) was added, and cells were filtered and collected using a 100-μm cell filter and centrifuged at 250*g*, 4 °C for 3 min. The supernatant was discarded, and 2 ml of red blood cell lysate (BioGenous, E238010) was added to dissolve red blood cells at room temperature for 3 min. Single-cell tissue suspension was obtained, and 1 ml of colon cancer organoid medium was added. The resuspended cells were gently blown and centrifuged again at 250*g* for 3 min. Then, extracellular matrix (ECM, which should be kept on ice for temporary storage and operation to prevent it from solidification) was added to mix the cells and quickly added into the center of a 48-well plate (20 μl per well). The culture plate was placed in a 37 °C, 5% CO₂ incubator for 15 to 25 min to coagulate the ECM. The colon cancer organoid medium (300 μl) was carefully added to each well and cultured in the constant temperature incubator [69,70].

### Platform of colon cancer organoid neutrophil micro-injection

Neutrophils were labeled using cell tracker (Sigma, Red PKH membrane label kit). The cell culture dish was used to prepare the microinjection dish. The organoids were carefully separated with a syringe needle and added into the organoid culture droplets. The culture dish was covered with mineral oil to prevent the organoid droplets from spreading. The inverted microscope was adjusted with a micro-syringe, and a holding pipette and an injection pipette (5 μm in diameter) were installed. A small amount of culture solution was then inhaled into the 2 needles to maintain pressure balance. Neutrophils were aspirated using the injection pipette, the organoids were fixed in the droplets with a holding pipette, and the injection pipette was adjusted to the same plane as the holding pipette. The pressure of the injection needle was increased, and neutrophils (1 × 10³) were injected into the organoid. The injected organoids were transferred to a new culture dish with the Pasteur pipette and cultured for 48 h.

### Construction of 3D-printed cell culture models

A 0.2% photoinitiator LAP (lithium phenyl-2,4,6-trimethylbenzoylphosphinate, Abmole, 85073-19-4) solution was prepared using sterile PBS and stirred at 37 °C in darkness for 3 h to ensure complete dissolution. A 10% Gelma was then prepared using the LAP solution, rotated, and stirred at 37 °C in dark conditions for 1 h. The resulting completely dissolved gel was filtered in a 0.45-μm filter. Tumor cells were prelabeled using a cell membrane tracer, diluted (1:1,000), incubated for 30 min at 37 °C, and washed twice with PBS. The prepared solution was then thoroughly mixed with the labeled tumor cells, ensuring no air bubbles, and gel clusters of specific shapes and sizes were formed. The gel was cross-linked by irradiating with 405-nm ultraviolet light for 30 s at a distance of 10 cm, followed by the addition of a culture medium. After model construction, the 3D tumor cell model was incubated overnight in a constant temperature cell incubator. After 24 h, neutrophils, labeled similarly with a membrane tracer, were added for coculture experiments [71].

### Neutrophil phagocytosis assay

Neutrophil phagocytic ability was detected by a pHrodo *E. coli* BioParticles Phagocytosis kit (Thermo Fisher Scientific, P35360). Neutrophils (1 × 10^6^) were cocultured with 20 μl of fluorescently labeled *E. coli* bioparticle solution in a 37 °C/5% CO_2_ incubator for 0.5 h. The results were observed in the OLYMPUS IX73 microscope and analyzed by ImageJ.

### Clinical colon cancer sample collection

With approval from the Medical Ethics Committee of Suzhou Municipal Hospital and informed consent from patients, colon tissues were collected from patients who underwent surgery at the Department of General Surgery of the same hospital between 2022 and 2023. Patients with combined severe infections, major trauma, immune disorders, neutropenia, or hematologic disorders were excluded. The study included 10 patients: 4 in the T1 stage and 6 in the T4 stage. All tissues were confirmed as colonic adenocarcinoma by H&E staining by the pathology department.

### Subcutaneous tumorigenesis and measurement of tumor volume and weight in mice

Female C57BL/6J laboratory mice aged 5 to 6 weeks were selected to establish the tumor model and were raised under specific pathogen-free (SPF) conditions. Anesthesia was induced by isoflurane with an inhalation anesthesia machine (RWD, R660), followed by shaving and disinfection of the tumor inoculation site. Colon cancer MC38 cell concentration was adjusted to 2 × 10^7^ cells/ml, and each mouse was inoculated with 100 μl of cell suspension subcutaneously using a 30-gauge needle at a depth of approximately 1 cm. The cell suspension was placed on ice to mitigate the onset of tumor cell apoptosis, and the inoculation was controlled to be completed within half an hour. Additionally, local disinfection of the grafted skin site was performed. Subsequently, the mice were returned to their cages for observation. Tumor formation typically occurs within a week. Ethical guidelines stipulate that mouse tumors should not exceed 2 cm in any dimension, with a sufficient size not exceeding 1.5 cm. Once tumors reached a certain size within ethical bounds, the experiments were terminated, and the mice were euthanized by inhaling 40% CO_2_ for 2 min (FENGSHI Group, FSZZ-2A). The tumors were excised, photographed, and measured using a vernier caliper. Tumor volume (*V*) was calculated using the following formula: *V* = (length × width^2^)/2. Finally, tumor weight was weighed and recorded on an electronic balance.

### Modeling of neutrophil-cleared mice

Neutrophils were depleted by intraperitoneal injection of InVivoMAb anti-mouse Ly6G/Ly6C (Gr-1) (Bioxcell, BE0075) 24 h prior and consolidated every 5 d to make sure neutrophils complete clearance.

### NAT of aggressive dose of fresh neutrophils and peritumoral IL-8 injection

Due to the large quantity and accordant biological functions with peripheral blood neutrophils (Fig. 7), after conducting a biosafety evaluation of cell infusion (Fig. 8), we chose mouse bone marrow-derived neutrophils for experimentation. Purified fresh mouse neutrophils were resuspended in sterile saline at a concentration of 1 × 10^8^ cells/ml and injected into the peripheral blood circulation of mice via the tail vein (100 μl per mouse, i.e., a total of 1 × 10^7^ neutrophils, which is 2 times the number of circulating neutrophils in the peripheral blood of mice) [31,72]. IL-8 was diluted to a concentration of 500 ng/ml using sterile saline, and 100 μl was injected subcutaneously around the tumor at 3 sites per mouse.

### Tissue hyalinization

Tissues obtained after cardiac perfusion were placed in a configured degreasing solution and left overnight in a horizontal shaker at 37 °C. The degreasing solution was changed every 2 to 3 d, and the degree of tissue hyalinization was observed, usually completed within 10 to 14 d. The degreased tissue was placed on a hyalinization table for refractive index matching. Then, immunofluorescence staining was then performed on the transparent tissue. Biotin anti-mouse Ly6G antibody (BioLegend, 1A8) was used to label neutrophils, and CD31 (Abcam, ab281583) was used to label vessels in tumors. The stained tissue was embedded in gel solution using the sandwich method, ensuring no air bubbles, and cooled at 4 °C for 2 h. Finally, the gel samples were removed from the molds and photographed under a microscope.

### Statistical analysis

Statistical analysis was performed using GraphPad version 9.0 software. The data of 4 indicators of NSRI were analyzed by Shapiro–Wilk test and frequency distribution. Measurement data were presented as the mean ± standard deviation. Comparisons between the 2 groups were made using paired or unpaired *t* tests. Data comparisons among multiple groups were performed using one-way analysis of variance (ANOVA), followed by Tukey’s test for between-group comparisons. *P* < 0.05 was considered statistically significant.

## Ethical Approval

This study received approval from the Medical Ethics Committee of The Affiliated Suzhou Hospital of Nanjing Medical University (approval number: KL901390). Peripheral blood specimens were collected from the cubital veins of healthy voluntary drug-free donors who provided written informed consent before participating. All experimental procedures were conducted in strict compliance with approved guidelines. The animal experiments were approved by the Suzhou Institute of Systems Medicine (no. ISM-IACUC-0171-R). All experimental procedures involving mice were carried out in strict accordance with the recommendations outlined in the Guide for the Care and Use of Laboratory Animals of the National Institutes of Health.

## Data Availability

The datasets used and analyzed during the current study are available from the corresponding author on reasonable request.
