# Supplementary material for: Highly Selective Exogenous Neutrophils Effectively Inhibit Growth of Colon Cancer under the Guidance of Precision Navigation
Source: Research (Wash D C). 2025 Sep 16;8:0894. doi: 10.34133/research.0894 (PMC12437111; doi:10.34133/research.0894)
Supplement: Supplementary 1 — Materials and Methods Figs. S1 to S7 Movies S1 to S4 [file research.0894.f1.zip › Si.docx]

**Supplementary materials for**

**Highly selective exogenous neutrophils effectively inhibit growth of colon cancer under the guidance of precision navigation**

by

Yunxi Yang, Chuyu Li, Yiming Shao, Xiao Wen, Cheng Lu, Xi Gao, Yiwen Mei, Bingwei Sun^*^

**^*^** **Corresponding author**

Email: sunbinwe@hotmail.com

**This file includes:**

Supporting Materials and Methods;

Supplementary figures 1-7;

Supplementary videos 1-4.

**Supporting Materials and Methods**

***Cell culture and passage***

Neutrophils and colon cancer cells were cultured in 1640 medium and DMEM containing 10% FBS and 1% double-antibody, respectively. Colon cancer cells - HT29 (FUHENG BIOLOGY, FH0024), HCT116 (FUHENG BIOLOGY, FH0027), SW480 (FUHENG BIOLOGY, FH0022) and MC38 (FUHENG BIOLOGY, FH0644) were passaged at 80% confluence. Following medium removal, samples were washed twice with sterile PBS. Subsequently, the cells were digested using 0.25% trypsin at 37°C for approximately 1 min. Upon visual confirmation of cell detachment, trypsin digestion was aborted by adding three times the volume of fresh medium, followed by repeated gentle agitation. Total cells were obtained by centrifugation and passaged accordingly. All the colon cancer cells grew well (Supplementary figure 2), the cell activity was investigated by Cell-Counting-Kit-8 (CCK8) detection.

***Direct cell co-culture experiment***

Tumour cells were pre-inoculated at a density of 2×10^5^ cells/well and incubated for 4 hours until attachment. In the cytochalasin B (cytb) intervention group, neutrophils were pre-treated with 100 nM cytb for 0.5 hours, then washed with PBS, and the cells were resuspended in the 1640 culture medium (10% FBS) for subsequent experiments. Neutrophils were subsequently added in proportion and co-cultured for 24 hours before observation and analysis. Cells were observed and photographed under microscope, scanning electron microscopy (SEM) and transmission electron microscope (TEM).

***Indirect cell co-culture experiment***

A Transwell system with 0.4μm pore size chambers were employed for co-culture experiments. Tumour cells were seeded in the lower chamber at a density of 2×10^5^ cells/well and allowed to attach for 4 hours. Neutrophils were added to the upper chamber in proportion. After 24 hours of indirect culture, each index was assessed.

***Transwell chemotaxis assay of neutrophils***

Transwells with 3 μm pore size were filled with 500 μL each of culture medium (containing 10% FBS), IL-8, tumour cells and tumour cell supernatant. Subsequently, 100 μL of mouse neutrophils resuspended in serum-free medium was added to the upper chamber (5×10^5^ cells) and incubated at 37℃ and 5%CO_2_. Following a 4-hour incubation, the internal test culture solution was removed from the Transwell chamber, washed twice with PBS and fixed with 4% paraformaldehyde solution for 30 min at room temperature. Then, the cells were stained with crystal violet solution for 20 min. Subsequently, the chamber was washed with PBS, placed on slides, and observed under an inverted microscope. The chemotaxis cells were photographed from five fields of view from each group. After photographing, the images were counted and quantitatively analysed using ImageJ software.

***Cell immunofluorescence assay***

After co-culture, the supernatant from the 3D cell model was aspirated, and cells were fixed with 4% paraformaldehyde for 30 min at room temperature. Cells were then permeabilised with Triton solution for 30 min and blocked with 5% BSA solution for 10 min. After PBST washing, cells were incubated with NE and GSDMD primary antibody overnight at 4℃ on a shaker. The primary antibody solution (recyclable) was aspirated and the corresponding fluorescent secondary antibody was added and incubated at room temperature in dark conditions for 1 h. F-actin staining solution was then added and incubated at 37°C in dark condition for 1 h. Subsequently, cell clusters were then sealed with a DAPI sealer and confocal images were taken after 10 min. Neutrophils of mice were stimulated by LPS (10 μg/mL) for 4h, NETs were stained and labelled with SYTOXGREEN. What’s more, neutrophils and *E.coli (RFP)* were photographed for phagocytosis after being co-cultured for 2 hours.

***NE content detection by ELISA***

Samples preserved at -80℃ were thawed overnight at 4℃. The ELISA procedure was conducted following the instructions (Abcam ab270204, ab252356). Finally, absorbance was detected at 450 nm using a microplate reader. The corresponding concentration of each well was calculated according to the standard curve and grouped for statistical analysis.

***Preparation of tumour single-cell suspension***

The extracted mouse tumours were cut into 1 mm diameter pieces, treated with 0.25% trypsin, and placed in a 37℃ shaker for 30 mins for gentle digestion. The digested tissue was then sieved through a 100 μm cell sieve to obtain single cells and supernatant for subsequent experiments.

***Detection of flow cytometric indexes***

Flow cytometric analysis was performed on 1×10^6^ cells from prepared tumour single-cell suspensions. Neutrophil percentages and CD54, CXCR4 expression were assessed through staining of Ly6G (BioLegend, 127614) and CD54 (BioLegend, 353108, 116105), CXCR4 (BioLegend, 146508). Subsequently, NETs content in intratumoral neutrophils was determined using double staining of Ly6G and SYTOXGREEN. Plasma inflammatory indexes in mice were measured using the BD CBA kit. Finally, the generation of reactive oxygen species (ROS) produced by neutrophils was labelled and detected by DCFH-DA.

***Mouse survival data***

Mice in the Control, 2-fold PBNC (peripheral blood neutrophil counts, PBNC) and 10-fold PBNC groups were monitored for one week, recording mobility, appetite, body weight, mental status, and survival. Subsequently, survival rates were calculated based on the recorded data.

***HE staining of tissues***

Paraffin sections were cleaned with distilled water and the tissue was fixed with alum-hematoxylin for 5 min for nuclear staining. Subsequently, the sections were neutralised using 0.3% acidic alcohol until the background appeared colourless. The slices were then immersed in eosin for 2 min, with excess eosin rinsed off with running tap water. Finally, the slices were dehydrated, cleaned, covered with clean coverslips, and photographed.

***Mouse cardiac perfusion***

Mice were injected intraperitoneally with 2% pentobarbital sodium aesthetic. Once anaesthetized, mice were fixed on the operating table with the abdomen facing up. A transverse incision was made in the abdomen, which was gradually expanded cephalad along the lateral abdominal wall to expose the abdominal and thoracic cavities and facilitate the perfusion process. The diaphragm was carefully clipped at the lower end of the xiphoid process and enlarged to both sides. Subsequently, the ribs on both sides were clipped, and the incision was expanded cephalad until the atria were exposed. The mouse raphe was clamped, and the thorax was turned outward to immobilise and fully expose the heart. The left ventricle was punctured with a 1-mL hypodermic needle, and blood was allowed to flow out by clipping the right auricle. Subsequently, sterile saline was slowly instilled until the outflow from the right auricle appeared clear and the liver, lungs, and mucous membranes turned white. Subsequently, 4% paraformaldehyde was injected until the mouse exhibited tails cocking and muscle twitching, indicating a successful perfusion. The perfusion rate was then slowed until the perfusion was completed, leaving the mouse in a rigid state. The perfusion apparatus was then removed, and the mouse body was transferred to a clean operating tray for tissue isolation. Finally, the obtained specimens were placed in 4% paraformaldehyde and stored in a 4°C medical refrigerator.

**Supplementary figures 1-7**


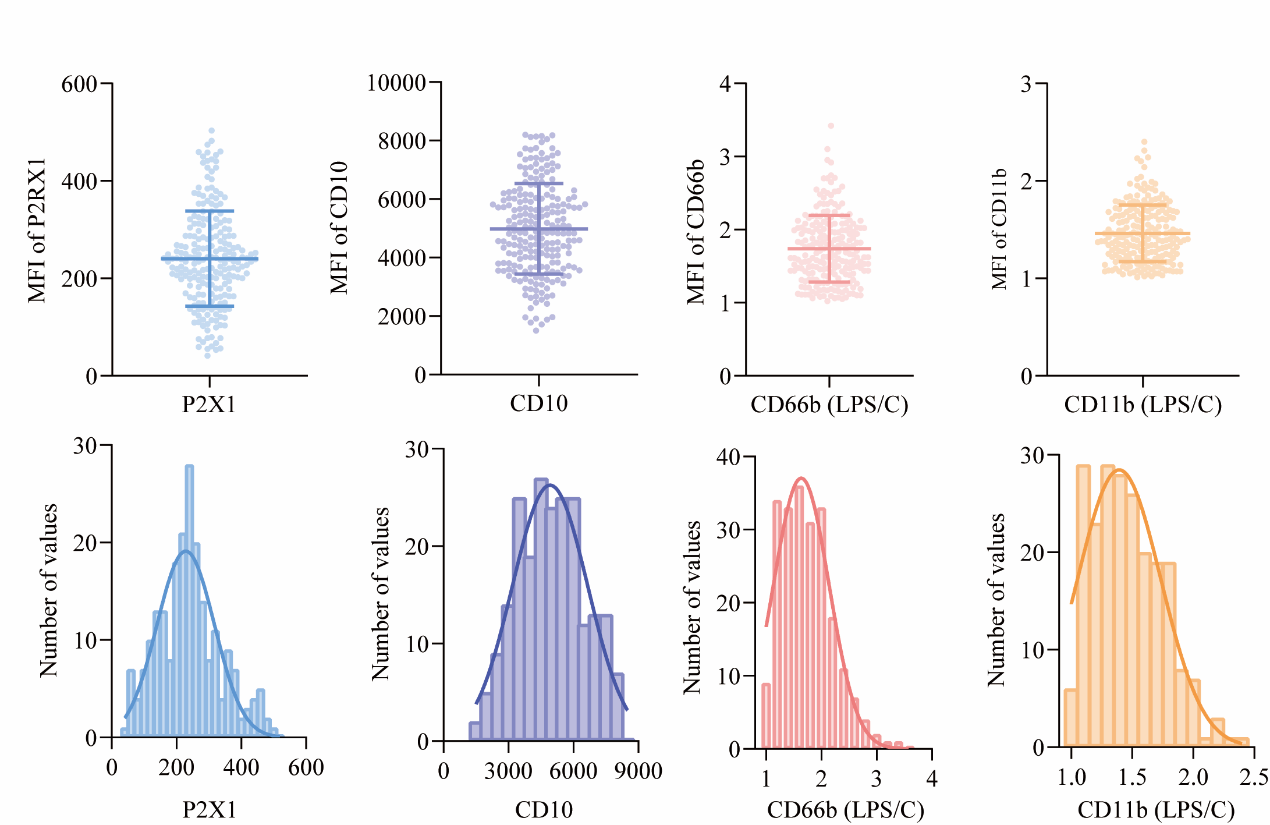


**Supplementary figure 1. Data distribution of indicators of PSRI**


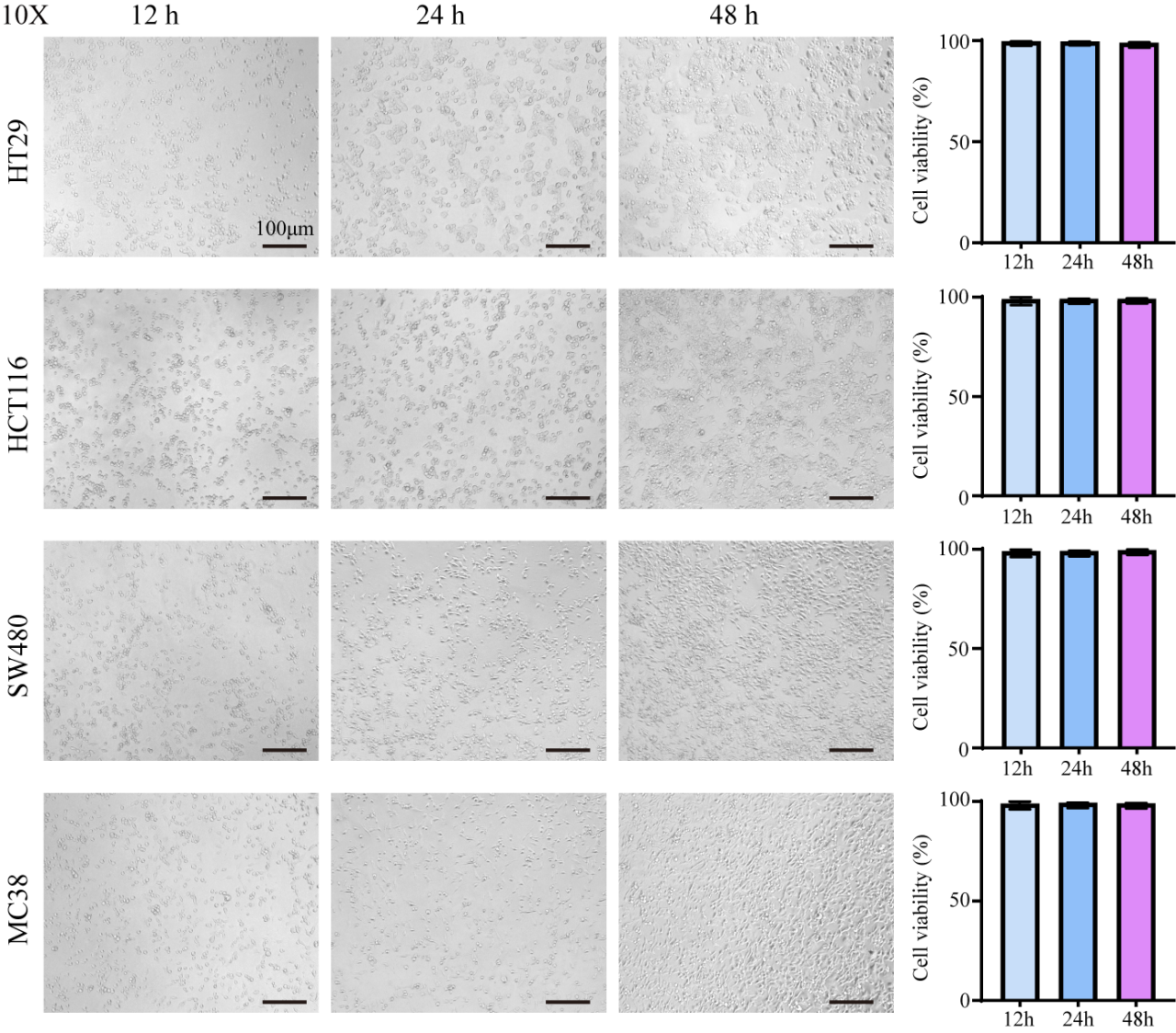
**Supplementary figure 2. The growth status of colon cancer cells**

Human colon cancer cell lines (HT29, HCT116 and SW480) and murine colon cancer cell line (MC38) grew well (scale bar, 100 μm) and the cell activity was higher than 97% at various stage of growth (12h, 24h and 48h).


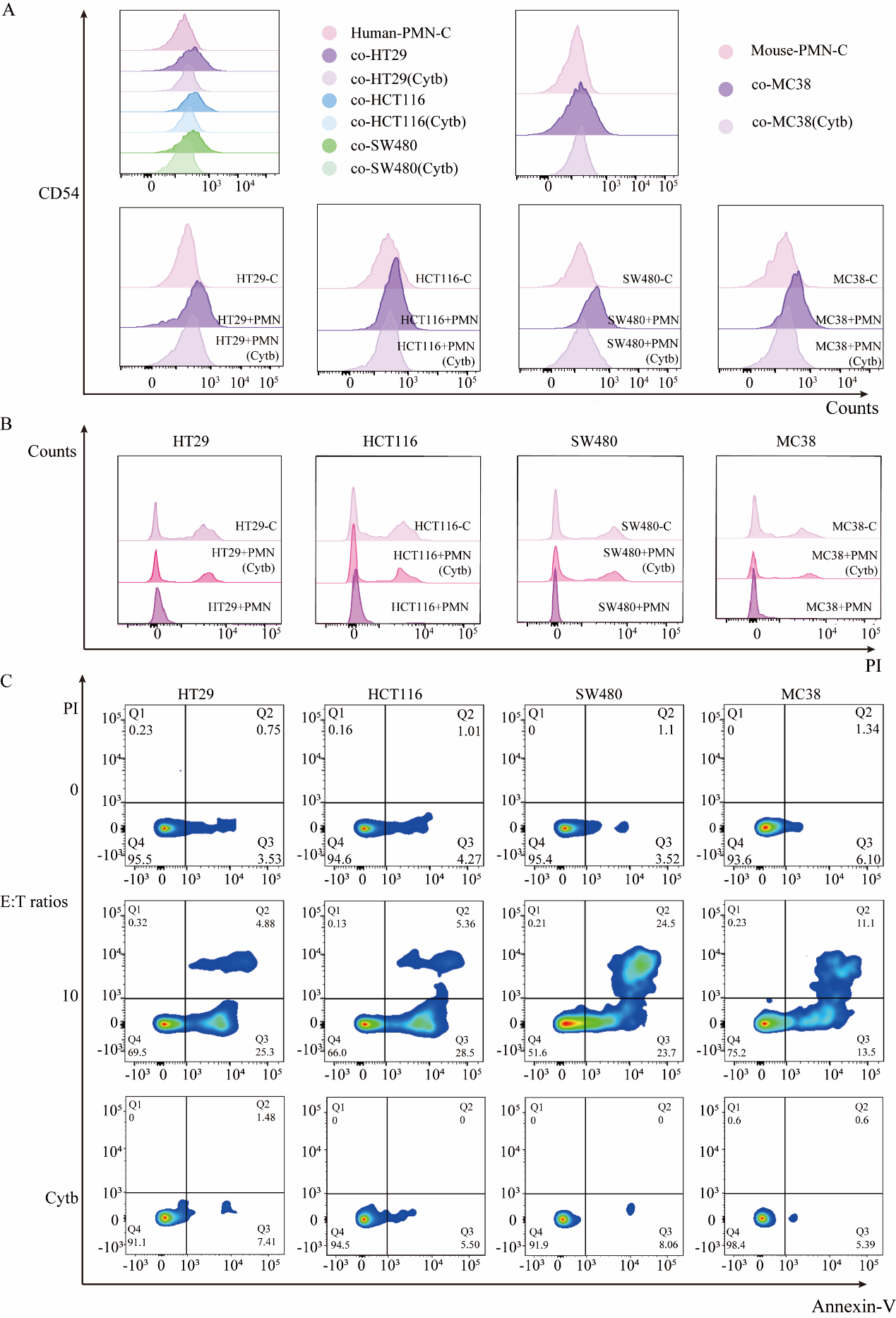


**Supplementary figure 3. The growth status of colon cancer cells after co-culture with neutrophils**

Flow cytometry patterns of CD54 expression (A), cell cycle (B) and apoptosis (C) of colon cancer cells after co-culture with neutrophils.

The expression of CD54 significantly increased in neutrophils and colon cancer cells (Supplementary figure 3A). Colon cancer cells exhibited pronounced G0/G1 phase arrest (Supplementary figure 3B) and increased apoptosis (Supplementary figure 3C) after co-culture. These results were not observed in co-cultures of colon cancer cells with Cytb- intervened neutrophils.


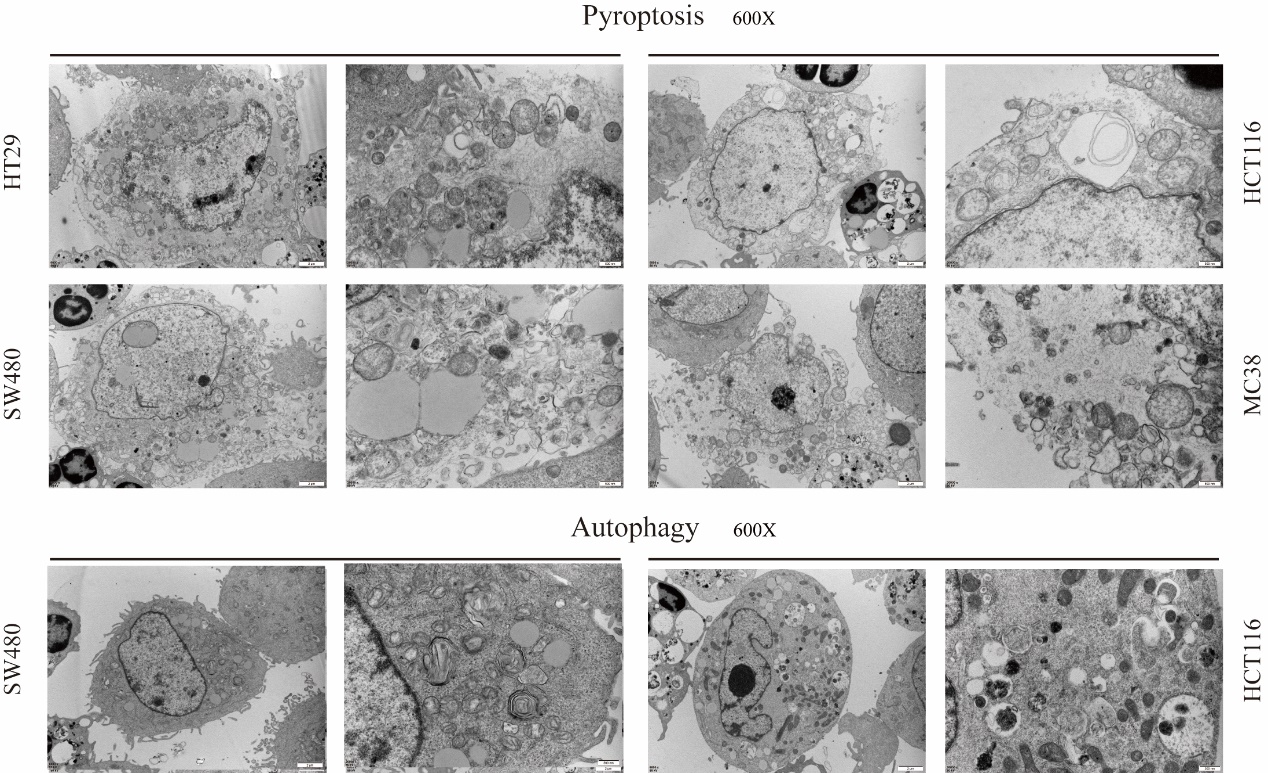


**Supplementary figure 4. Pyroptosis and autophagy of colon cancer cells after co-culture**

In addition to apoptosis, the swelling of mitochondria and endoplasmic reticulum，and the formation of vesicles-like protrusion - pyroptosis corpuscles were also observed in colon cancer cells. Phagocytic vesicles and autophagosomes were as well observed in HCT116 and SW480 cells, indicating the formation of autophagy.


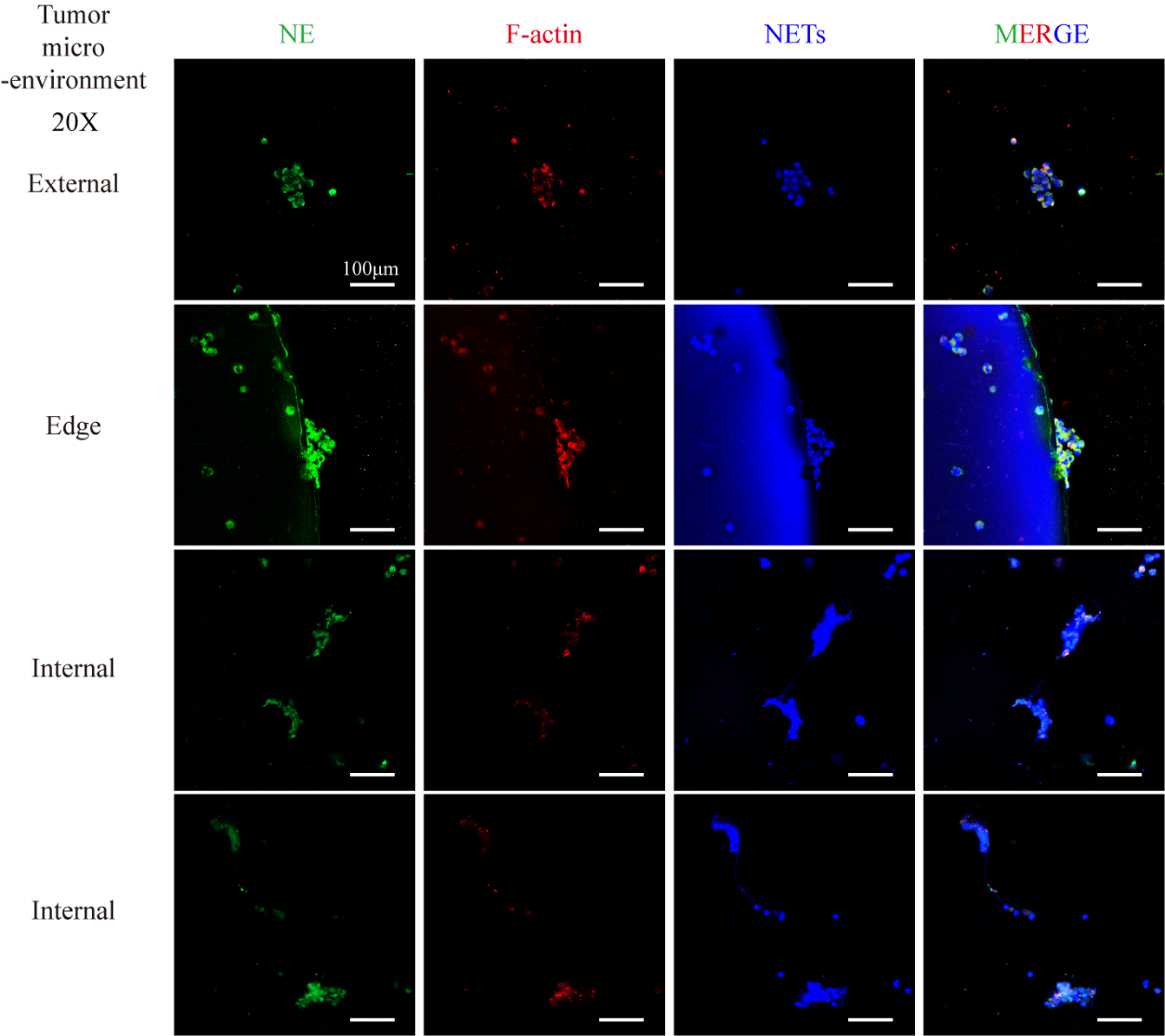


**Supplementary figure 5. Neutrophil infiltrate into the tumour microenvironment**

In the outside of the tumour microenvironment, neutrophils are regular round, NE is in the cytoplasm, skeleton and DNA are intact. As the neutrophils gradually enter the inside of the microenvironment, the morphology, nucleus and skeleton of the neutrophils are changed, NETs formation and the NE is released from the cytoplasm (Scale bar, 100 μm).


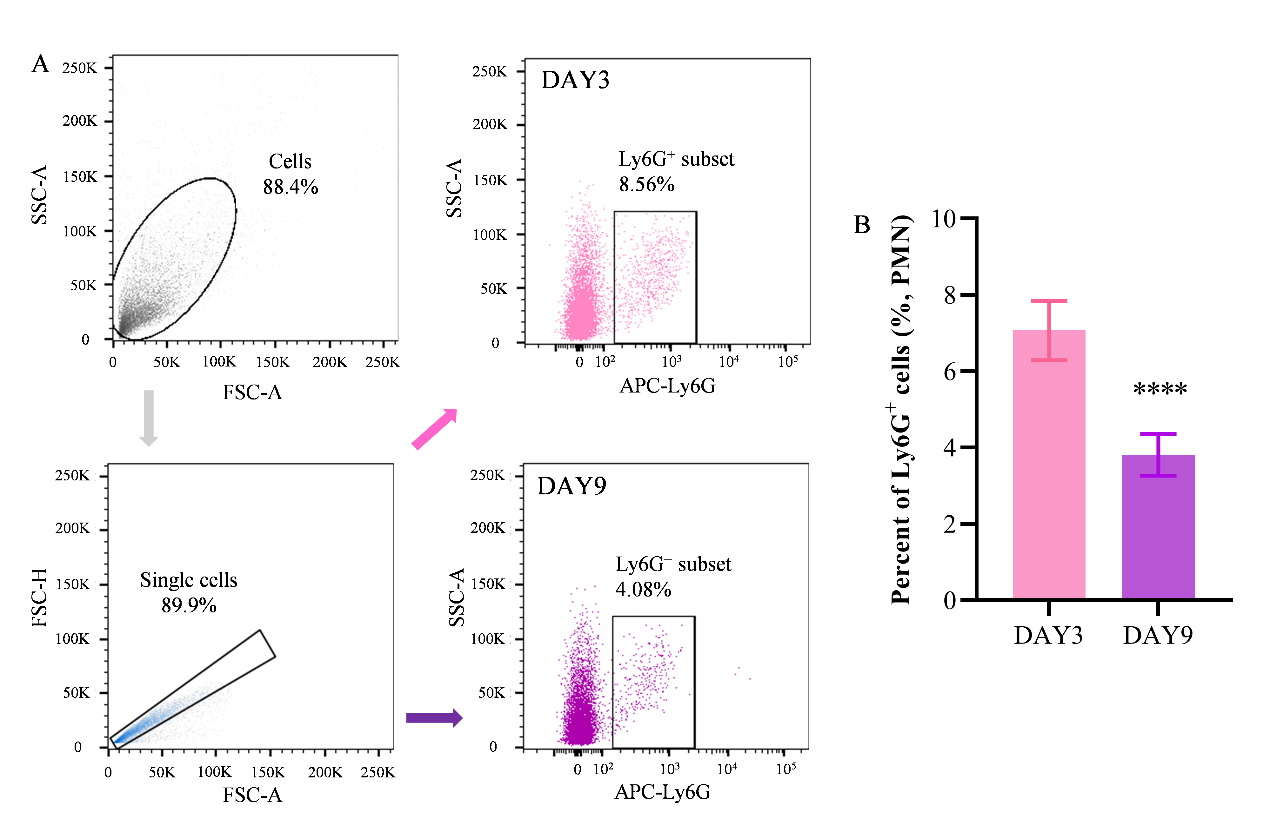


**Supplementary figure 6. Neutrophils exhibit significant infiltration during the early stages of colon cancer.**

(A) Flow cytometry analysis and representative gating strategy of single-cell suspension preparation from tumor tissues; (B) Analysis of neutrophil proportions in subcutaneous tumors at Day 3 and Day 9.

To analyze the cellular composition of subcutaneous tumors formed at Day 3 and Day 9, we prepared single-cell suspensions from tumor tissues and identified neutrophils using Ly6G staining (Supplementary Figure 6A). Flow cytometry analysis revealed significantly higher neutrophil infiltration in Day 3 tumors compared to Day 9 (Supplementary Figure 6B), consistent with H&E staining results (Figure 9A). These findings suggest that dynamic changes in tumor-associated neutrophil levels may correlate with tumor growth progression.


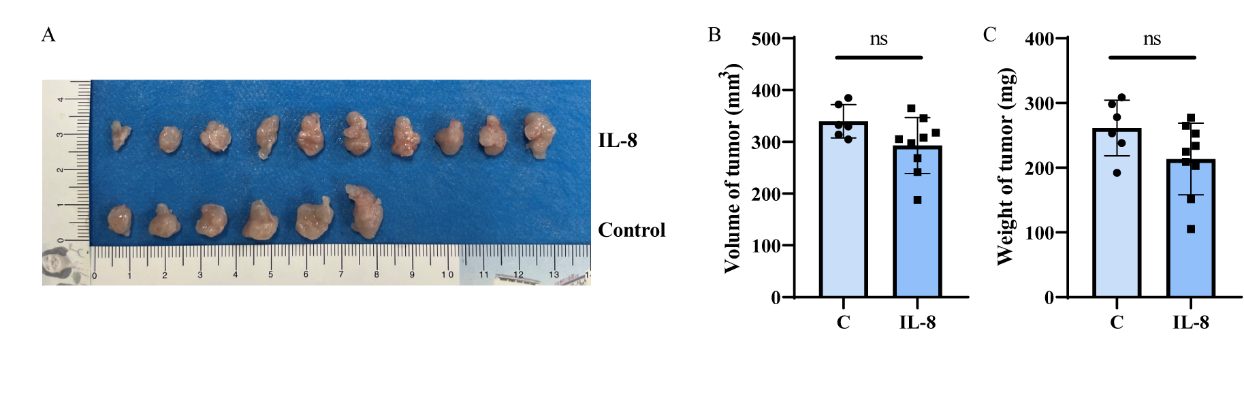


**Supplementary figure 7. Subcutaneous administration of IL-8 did not exert a statistically significant impact on tumor growth**

(A) Tumor growth kinetics in control versus IL-8-treated groups (peri-tumoral injection); (B) Comparative tumor volume analysis; (C) Tumor weight assessment at endpoint.

To investigate the potential inhibitory effect of IL-8 on growth of colorectal tumors, recombinant IL-8 (500 ng/mL, 100 μL) was administered via peri-tumoral subcutaneous injection. Beginning post-tumor establishment, treatments were delivered every 48 hours for a total of five doses. Tumor dimensions (length and width) were serially monitored using calliper measurements (Supplementary Figure 7A). The analyses revealed that no significant differences in final tumor volume or weight between the IL-8-treated and control groups (Supplementary Figures 7B–C). These findings demonstrate that localized delivery of exogenous IL-8 did not significantly alter the progression of colorectal tumor growth under these experimental conditions.

**Supplementary videos 1-4**


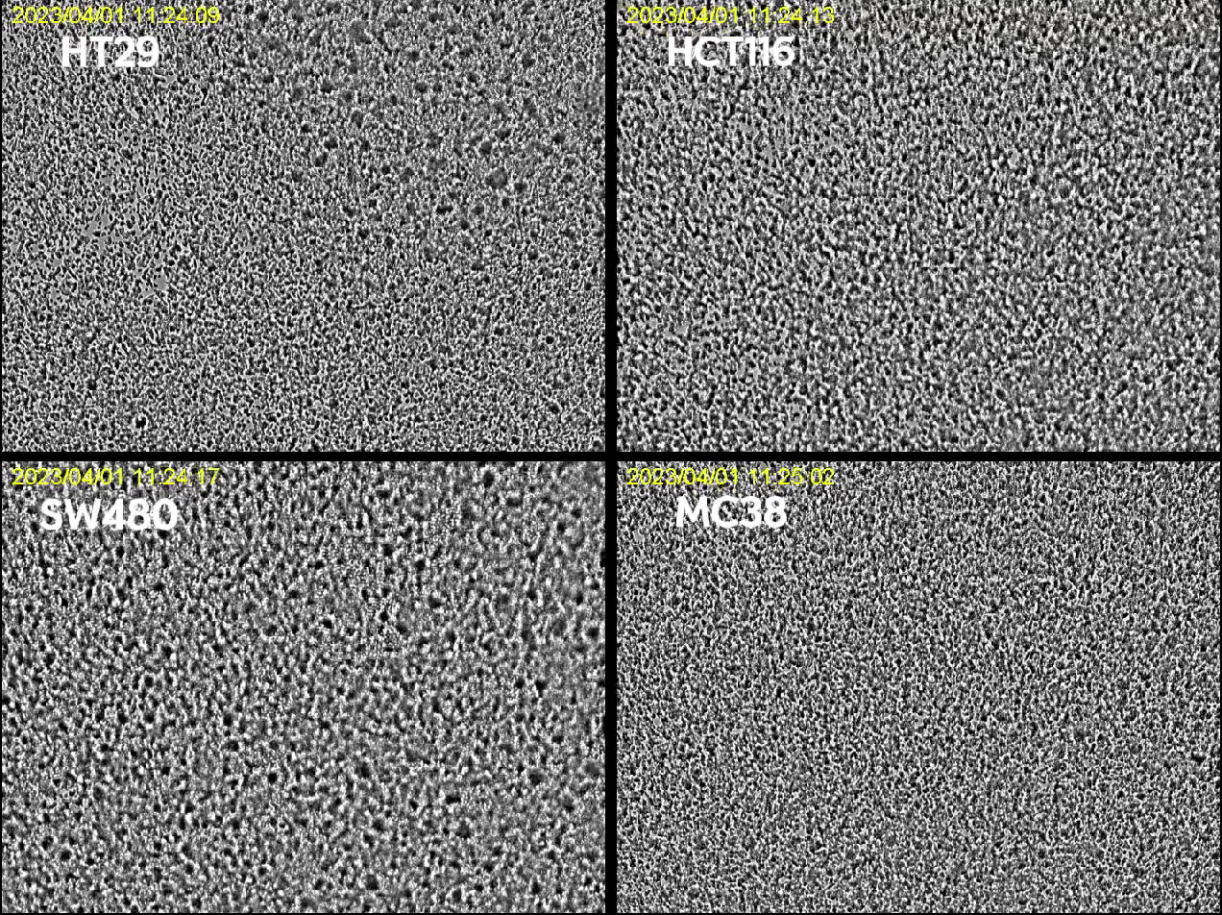


**Supplementary video 1. Dynamic observation of the growth of colon cancer cells in 64h co-culture with neutrophils**


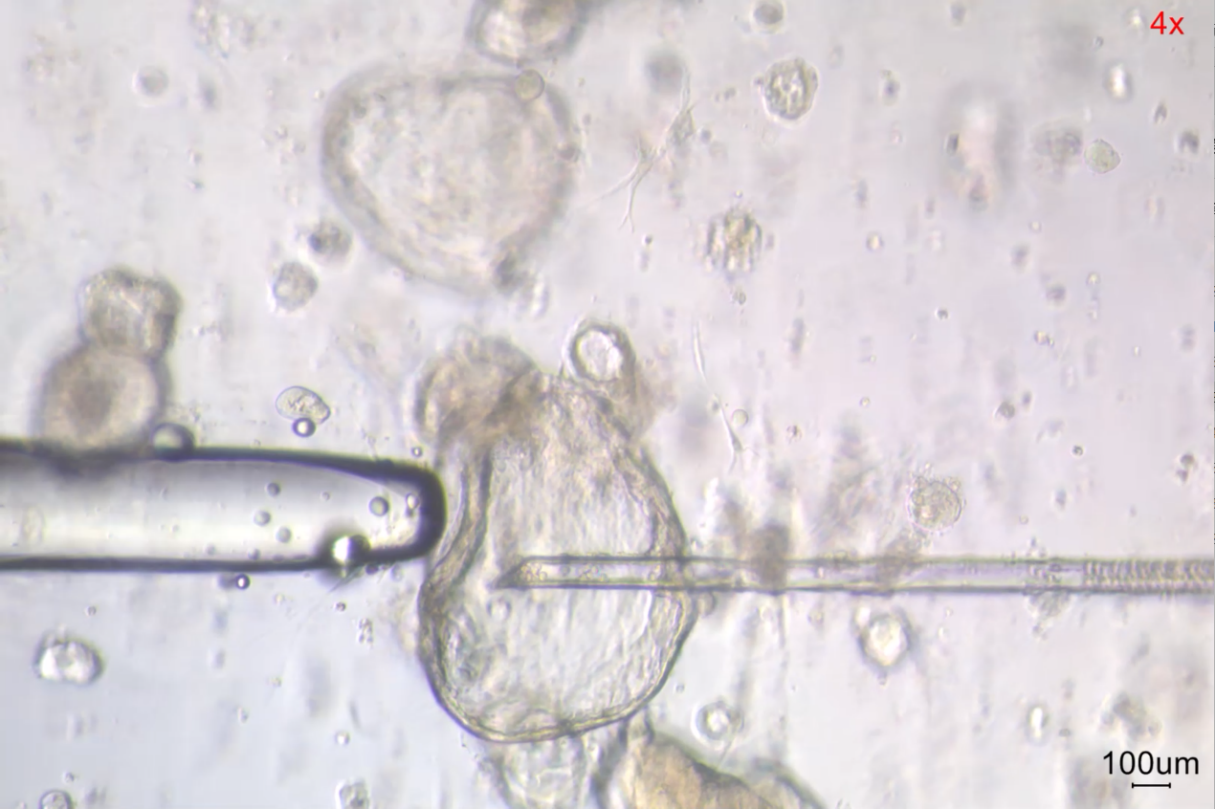


**Supplementary video 2. Colon cancer organoids neutrophils micro-injection**


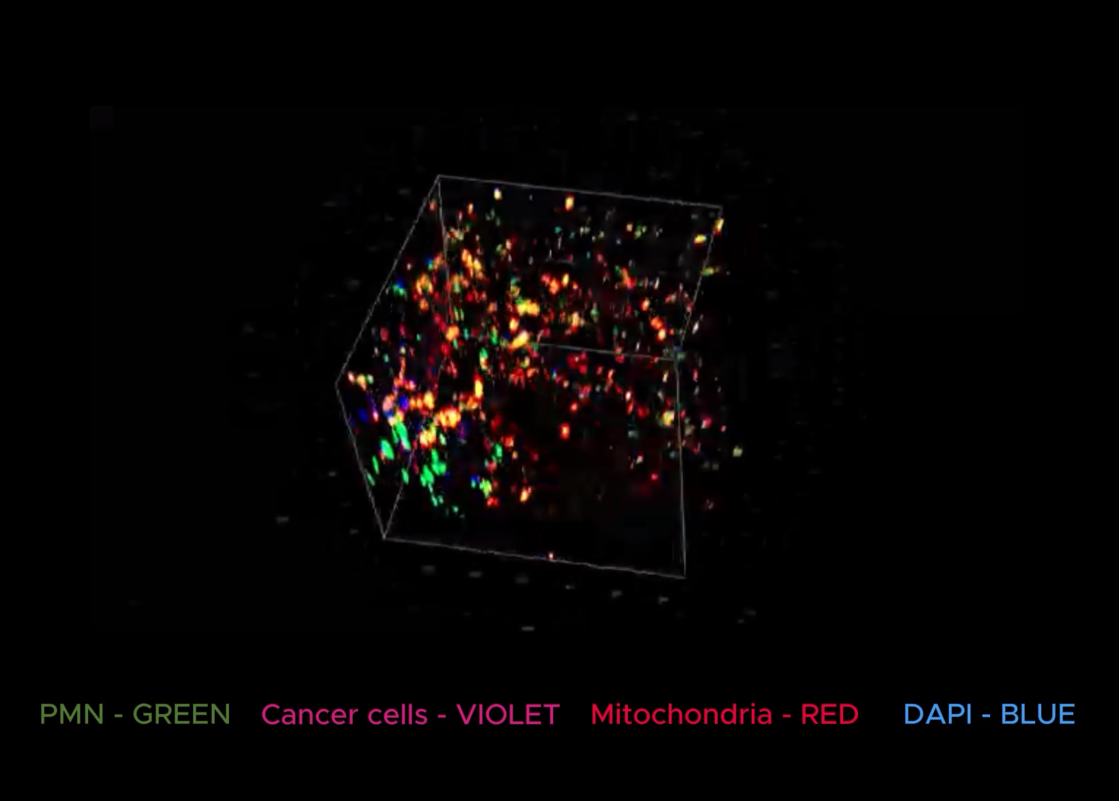


**Supplementary video 3. Stereoscopic imaging of 3D printing co-culture model**


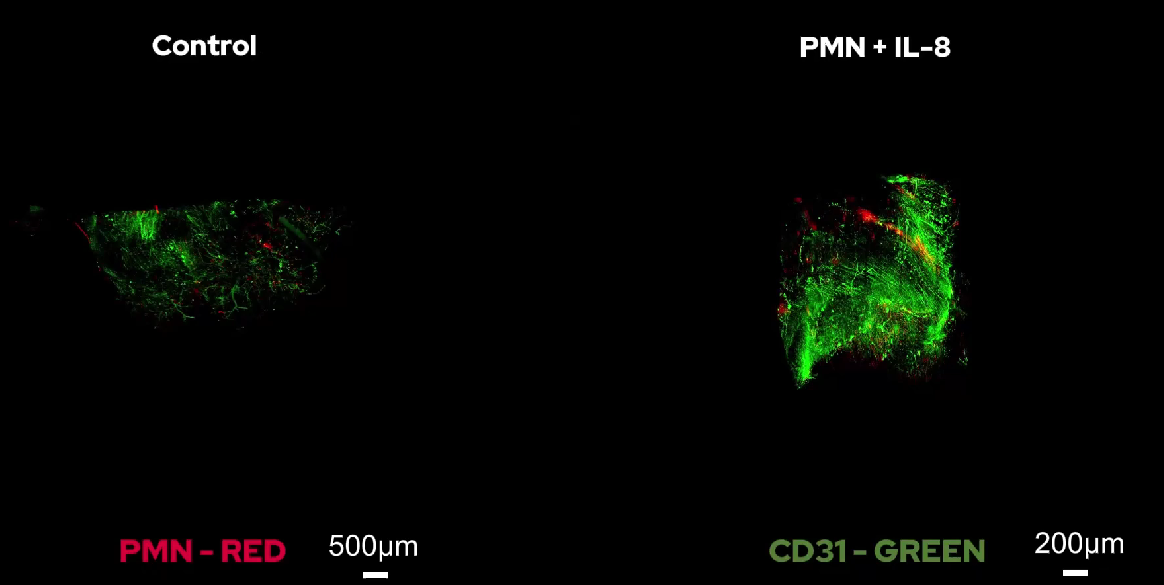


**Supplementary video 4. Stereoscopic imaging of transparent tumour tissues**
